# Supplementary material for: SAC1 regulates autophagosomal phosphatidylinositol-4-phosphate for xenophagy-directed bacterial clearance
Source: Cell Rep. Author manuscript; Available in PMC 2021 Aug 2. (PMC8327279; doi:10.1016/j.celrep.2021.109434)
Supplement: 1 [file NIHMS1728472-supplement-1.pdf]

Cell Reports, Volume *36*

Supplemental information

SAC1 regulates autophagosomal phosphatidylinositol-4-phosphate for xenophagy-directed bacterial clearance

Kai Liu, Lingjia Kong, Daniel B. Graham, Kimberly L. Carey, and Ramnik J. Xavier

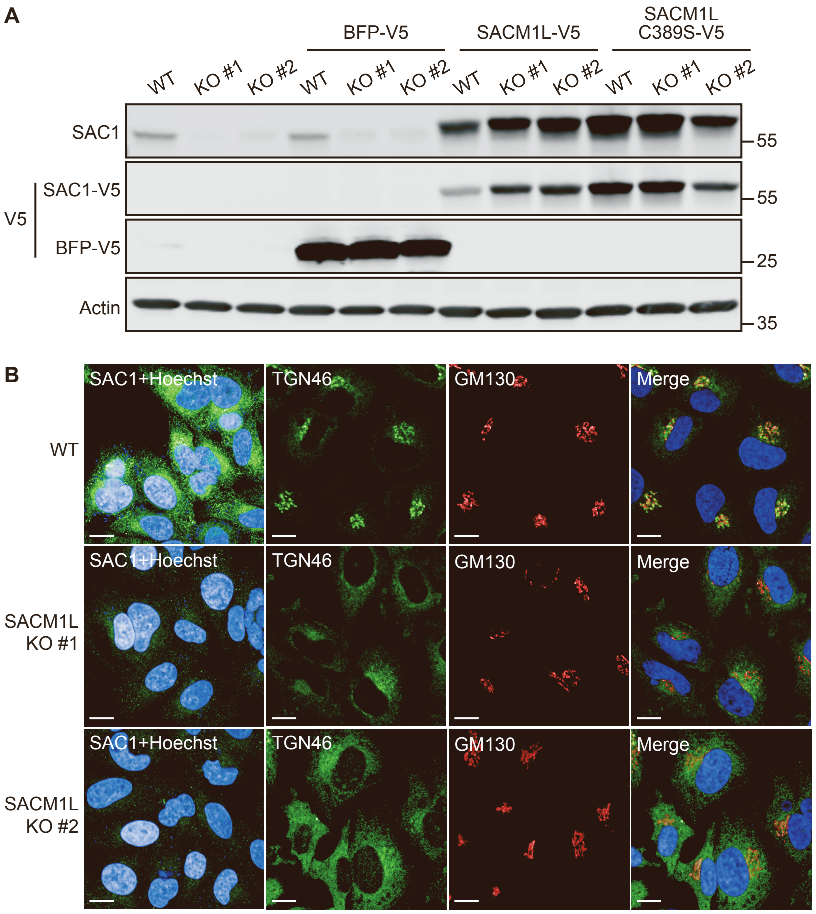

Figure S1. Validated SAC1-deficient cell lines have dispersed trans-Golgi network (TGN), Related to Figure 1. A) Representative immunoblot of WT and *SACM1L* KO cells with or without stable expression of V5-tagged BFP, SAC1 WT, or phosphatase inactive SAC1 C389S. Cells were reconstituted by lentiviral transduction and selected with puromycin for 7 days before the experiment. Endogenous and re-expressed SAC1 were detected with an antibody against SAC1. Recombinantly expressed SAC1 WT, SAC1 C389S, and BFP were detected with an antibody against V5. Actin served as a loading control. Molecular weight markers are shown on the right. **B)** Knockout of *SACM1L* causes dispersed Golgi morphology. In representative confocal images, SAC1+Hoechst images demonstrate cytoplasmic localization of SAC1 in WT cells. No SAC1 was detected in *SACM1L* KO cells. Golgi and TGN were detected with antibodies against GM130 and TGN46, respectively, in WT and *SACM1L* KO cells. Merged images of GM130, TGN46 and Hoechst are shown. Scale bars represent 20µm. For all quantifications, over 500 cells were analyzed. Data from three independent experiments were analyzed using ANOVA (mean ± SEM).

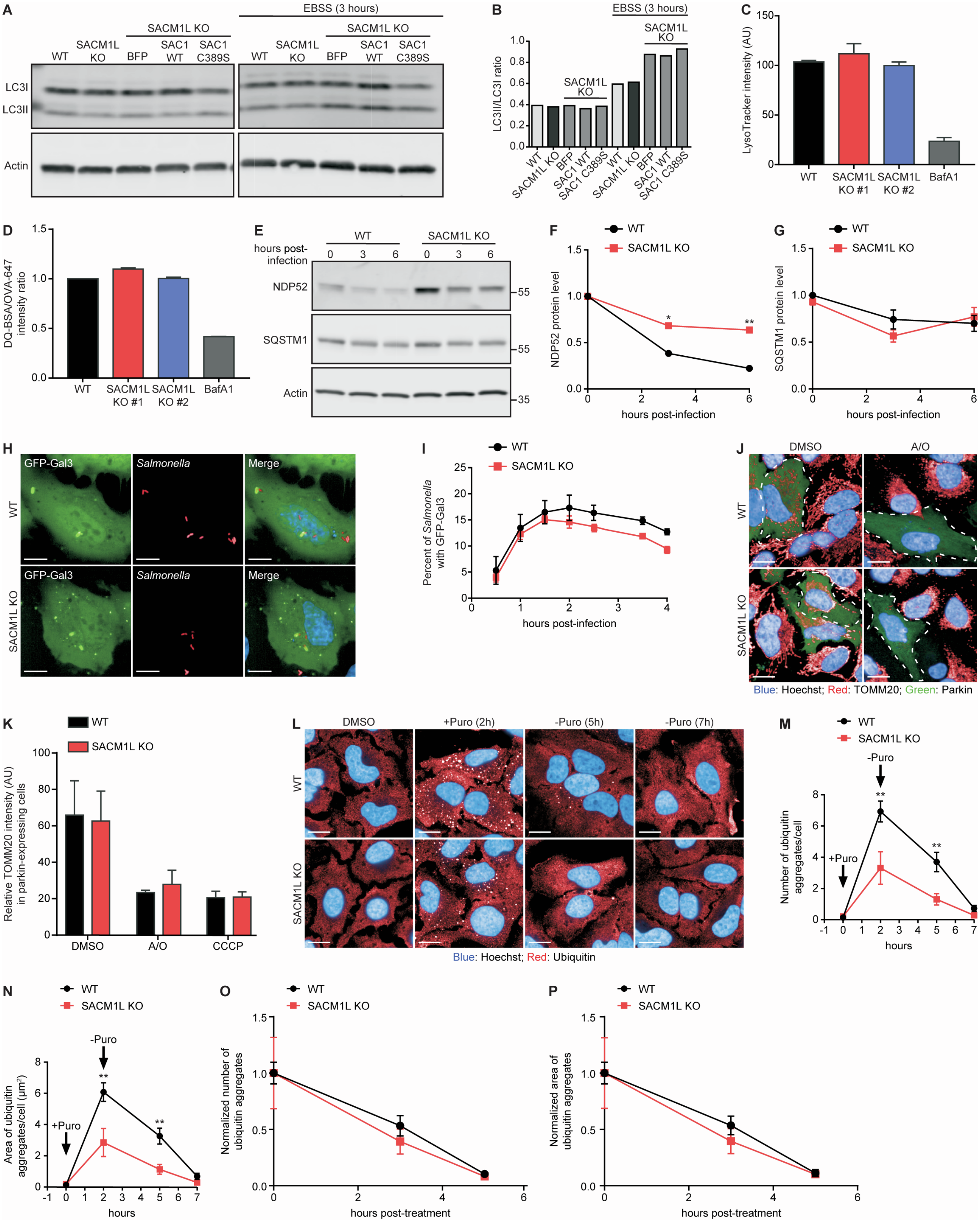

A
EBSS (3 hours)
SACM1L KO
WT
SACM1L KO
BFP
SAC1 WT
SAC1 C389S
LC3I
LC3II
Actin
B
EBSS (3 hours)
SACM1L KO
LC3II/LC3I ratio
1.0
0.8
0.6
0.4
0.2
0.0
WT
SACM1L KO
BFP
SAC1 WT
SAC1 C389S
C
LysoTracker intensity (AU)
150
100
50
0
WT
SACM1L KO #1
SACM1L KO #2
BafA1
D
DQ-BSA/OVA-647 intensity ratio
1.5
1.0
0.5
0.0
WT
SACM1L KO #1
SACM1L KO #2
BafA1
E
hours post-infection
WT
SACM1L KO
0
3
6
NDP52
SQSTM1
Actin
55
55
35
F
WT
SACM1L KO
NDP52 protein level
hours post-infection
G
WT
SACM1L KO
SQSTM1 protein level
hours post-infection
H
GFP-Gal3
Salmonella
Merge
WT
SACM1L KO
I
WT
SACM1L KO
Percent of Salmonella with GFP-Gal3
hours post-infection
J
DMSO
A/O
WT
SACM1L KO
Blue: Hoechst; Red: TOMM20; Green: Parkin
K
WT
SACM1L KO
Relative TOMM20 intensity (AU) in parkin-expressing cells
DMSO
A/O
CCCP
L
DMSO
+Puro (2h)
-Puro (5h)
-Puro (7h)
WT
SACM1L KO
Blue: Hoechst; Red: Ubiquitin
M
WT
SACM1L KO
-Puro
+Puro
Number of ubiquitin aggregates/cell
hours
N
WT
SACM1L KO
-Puro
+Puro
Area of ubiquitin aggregates/cell (µm²)
hours
O
WT
SACM1L KO
Normalized number of ubiquitin aggregates
hours post-treatment
P
WT
SACM1L KO
Normalized area of ubiquitin aggregates
hours post-treatment

Figure S2. Loss of SAC1 activity has no effect on bulk autophagy, mitophagy or aggrephagy, Related to Figure 2. A-B) Representative immunoblot (A) and quantification (B) of LC3 conversion in WT, *SACM1L* KO and reconstituted cells grown in complete or amino acid-deficient (EBSS) media for 3 hours to induce non-selective autophagy. Actin served as a loading control. **C-D)** *SACM1L* KO cells have normal lysosomal function. FACS quantification of LysoTracker intensity (AU, arbitrary units) (C) or DQ-Green BSA degradation activity (D) in WT and *SACM1L* KO cells. WT cells were treated with BafA1 (200ng/ml) for 4 hours as a control for defective lysosomal acidification and degradation. Cells in (D) were treated with DQ-BSA (10µg/ml), OVA-647 (5µg/ml) and EGF (50ng/ml) for 1 hour, washed then cultured for 30 minutes before FACS analysis. **E-G)** Delayed NDP52 degradation in *SACM1L* KO cells after *Salmonella* infection. Representative immunoblot (E) and quantification of NDP52 (F) and SQSTM1 (G) protein levels in WT and *SACM1L* KO cells at indicated time points post-infection. Protein levels were normalized to the start of infection for each genotype. Actin served as a loading control. **H-I)** SAC1 does not affect Galectin 3 dynamics on *Salmonella*-containing vacuoles. Representative confocal images (H) and quantification (I) of *Salmonella* associated with GFP-Gal3 in WT and *SACM1L* KO cells. Cells transiently expressing GFP-Gal3 were infected with DsRed-expressing *Salmonella*. Following gentamicin treatment, cells were imaged by live confocal microscopy. (H) Representative images at 1 hour post-infection. Scale bars represent 10µm. (I) Quantification of the percentage of *Salmonella* co-localized with GFP-Gal3 at indicated time points post-infection. **J-K)** Loss of SAC1 does not affect Parkin-dependent mitophagy. Representative confocal images (J) and quantification (K) of TOMM20 in WT and *SACM1L* KO cells transiently expressing mCherry-Parkin. Following transfection, cells were treated with DMSO, CCCP (20µM) or a combination of oligomycin (5µM) and antimycin A (2µM), indicated as A/O, for 24 hours. Images are pseudo-colored such that endogenous TOMM20, Parkin and Hoechst are shown in red, green and blue, respectively. Quantification represents intensity of TOMM20 signal in Parkin^{+} cells within the dashed lines in images (J). Scale bars represent 20µm. **L-P)** Loss of SAC1 does not affect aggrephagy. Representative confocal images (L) and quantification (M-P) of ubiquitin immunostaining in WT and *SACM1L* KO cells. Cells were treated with puromycin (5µM) for 2 hours to induce formation of protein aggregates, washed then cultured for 3 hours or 5 hours before immunostaining. Quantification represents the number (M) and area (N) of ubiquitin^{+} aggregates per cell at indicated time points. Normalized number (O) and area (P) of ubiquitin^{+} aggregates was determined by dividing the data at the indicated time point by the data after 2 hours of puromycin treatment. Scale bars represent 20µm. For all quantifications, over 200 cells were analyzed. Data from three independent experiments were analyzed using ANOVA (mean ± SEM). *$p<0.05$, **$p<0.01$.

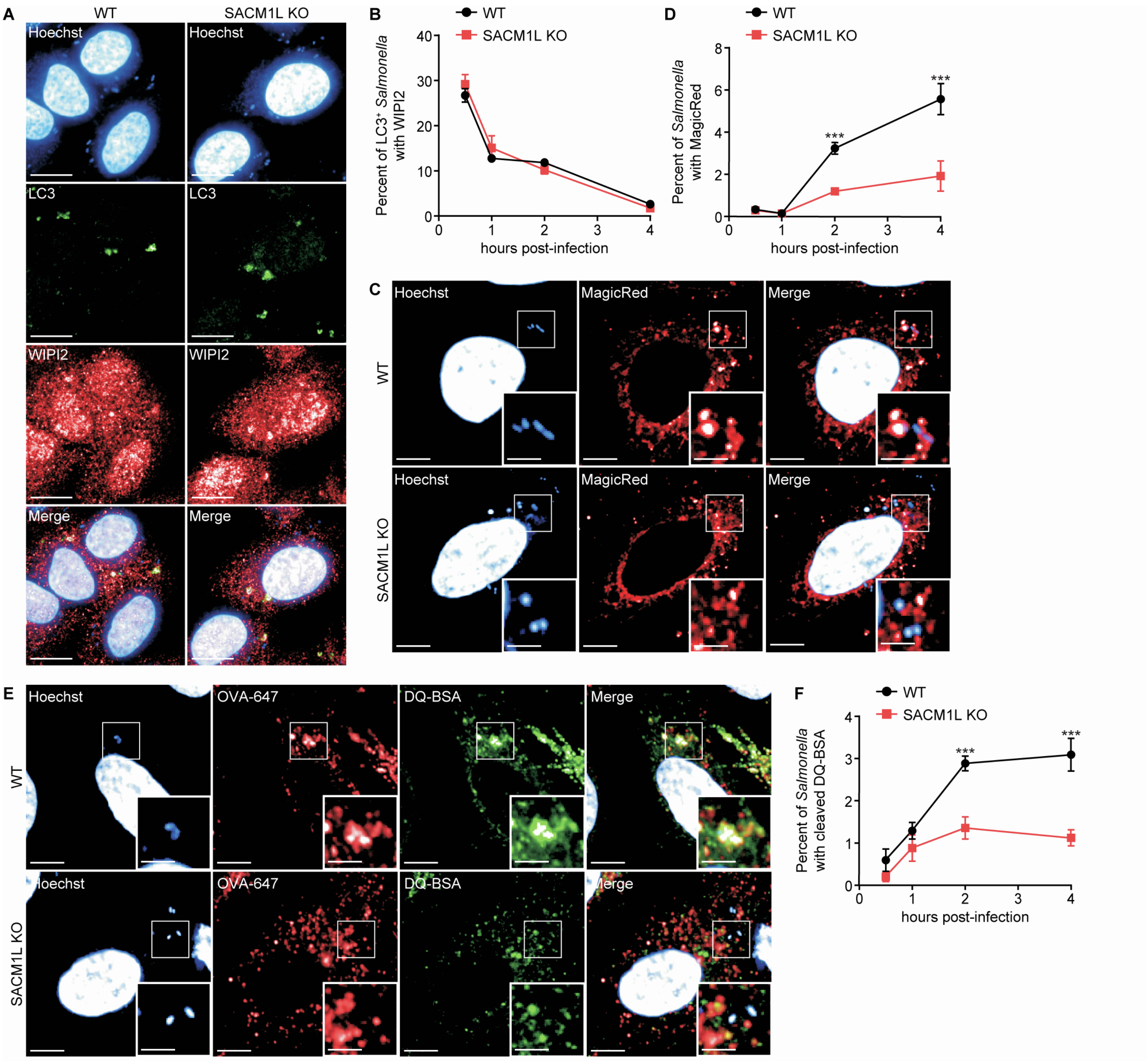

Figure S3. Loss of SAC1 delays delivery of lysosomal enzymes, not autophagosome closure, Related to Figure 3. A-B) WIPI2 recruitment and dissociation from *Salmonella*-containing autophagosomes are not affected by SAC1. Representative confocal images (A) and quantification (B) of *Salmonella* associated with WIPI2 in WT and *SACM1L* KO cells. Cells were fixed and imaged at indicated time points post-infection. A) Representative images of the infected cells at 1 hour post-infection. Scale bars represent 20μm. B) Percentage of $LC3^{+}$ *Salmonella* that are also $WIPI2^{+}$ at indicated time points post-infection. **C-D)** Delivery of cathepsin B to *Salmonella*-containing autophagosomes is delayed by SAC1 depletion. Representative confocal images (C) and quantification (D) of *Salmonella* associated with MagicRed in WT and *SACM1L* KO cells. Cells were pretreated with MagicRed for 30 minutes before infection, fixed and imaged at indicated time points post-infection. C) Representative images of the infected cells at 2 hours post-infection. Insets show magnified (2x) views of the boxed region in each image. Scale bars represent 10μm in full images and 5μm in insets. D) Percentage of $MagicRed^{+}$ *Salmonella* at indicated time points post-infection. **E-F)** Delivery of lysosome hydrolases to *Salmonella*-containing autophagosomes is delayed by SAC1 depletion. Representative confocal images (E) and quantification (F) of *Salmonella* associated with DQ-Green BSA and OVA-647 in WT and *SACM1L* KO cells. Cells were pretreated with DQ-Green BSA (10μg/ml) and OVA-647 (5μg/ml) for 1 hour before being infected, fixed and imaged at indicated time points post-infection. E) Representative images of the infected cells at 2 hours post-infection. Insets show magnified (2x) views of the boxed region in each image. Scale bars represent 10μm in full images and 5μm in insets. F) Percentage of DQ-Green BSA^{+} *Salmonella* at indicated time points post-infection. For all quantifications, over 500 cells were analyzed. Data from at least two independent experiments were analyzed using ANOVA (mean ± SEM). ***$p<0.001$.

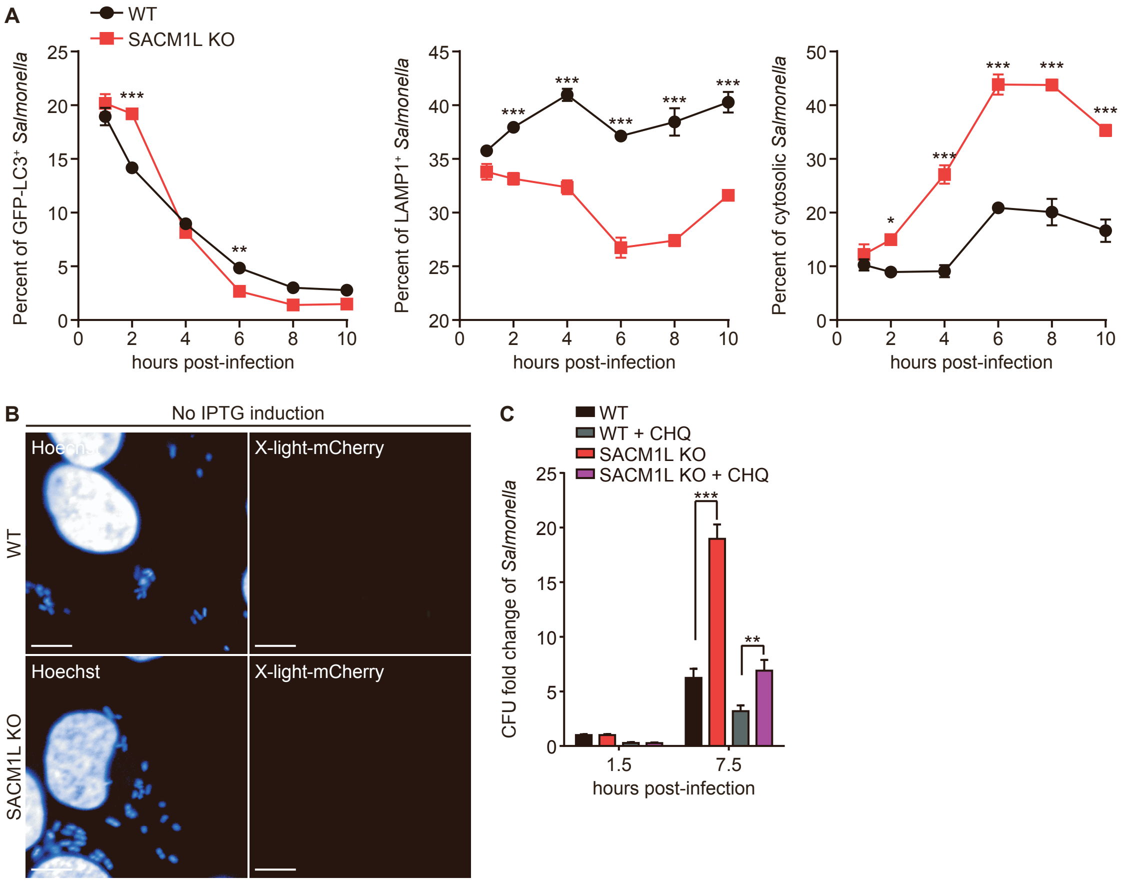

Figure S4. Delayed autophagosome-lysosomal fusion results in increased cytosolic bacteria, Related to Figure 4. A) Percentage of GFP-LC3^{+}, LAMP1^{+} or GFP-LC3^{-}LAMP1^{-} (cytosolic) *Salmonella* in WT and *SACM1L* KO cells at indicated time post-infection. Quantification of GFP-LC3^{-}LAMP1^{-} *Salmonella* populations excludes bacteria with LC3 or LAMP1 signal in proximity to make the data more precise. Over 5,000 bacteria were analyzed. **B)** Representative confocal images of *Salmonella* expressing x-light-mCherry without IPTG induction in WT and *SACM1L* KO cells at 6 hours post-infection. Scale bars represent 10μm. **C)** Contribution of non-SCV bacteria to increased intracellular replication in *SACM1L* KO cells. Infected cells were treated with 200 μM chloroquine (CHQ) for 1 hour prior to lysis and plating for CFU. Fold change of CFU was normalized to the 1.5-hour time point at indicated time points post-infection. For all quantifications, over 3,000 cells and 5,000 bacteria were analyzed. Data from at least three independent experiments were analyzed using ANOVA (mean ± SEM). *$p<0.05$, **$p<0.01$, ***$p<0.001$.

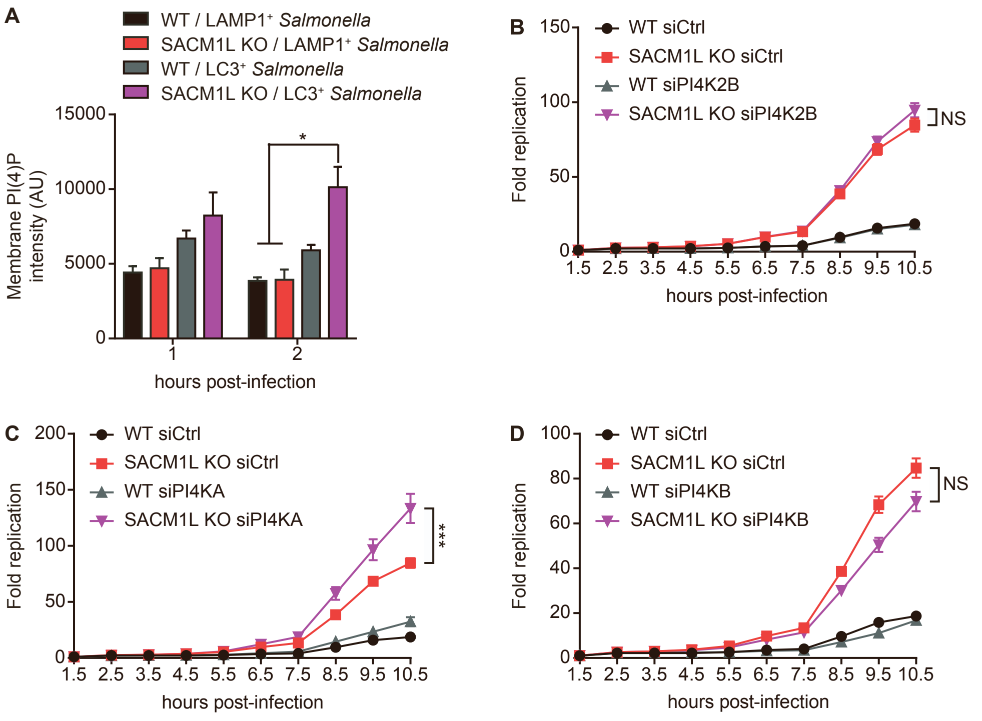

Figure S5. Defect in xenophagy due to loss of SAC1 is not rescued by kinase expression, Related to Figure 5 and Table S2. A) Quantification of the PI(4)P staining intensity (AU, arbitrary units) on $LC3^+$ or $LAMP1^+$ *Salmonella* in WT and *SACM1L* KO cells at indicated time points post-infection. **B-D)** For quantifications in knockdown experiments, WT or *SACM1L* KO cells were transfected with siRNA for control (Ctrl) or indicated genes. After 48 hours, cells were infected with *Salmonella* SL1344 expressing luciferase, and luciferase levels were measured over time. Bacterial replication was normalized to the baseline infection level. Knockdowns of PI4K2B (B), PI4KA (C) or PI4KB (D) do not affect *Salmonella* replication in WT or *SACM1L* KO cells. Data from at least two independent experiments were analyzed using ANOVA (mean ± SEM). *p<0.05, ***p<0.001. NS, not significant.

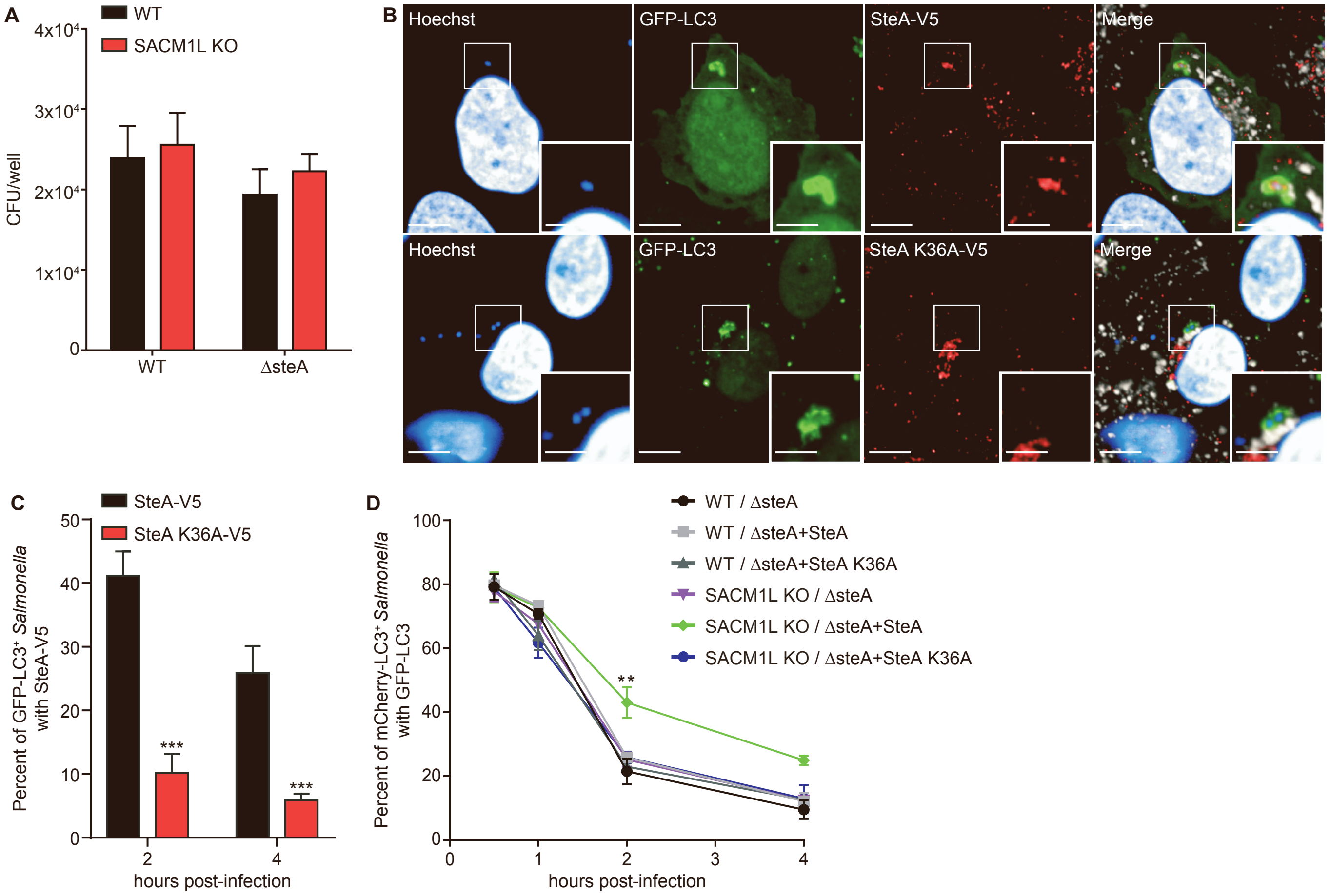

Figure S6. SteA PI(4)P binding is required to bind autophagosomes and block lysosomal fusion, Related to Figure 6. A) Neither loss of SAC1 nor *Salmonella* SteA affects bacterial uptake. Quantification of CFU/well of WT or Δ*steA Salmonella* at 1 hour after infection of WT or *SACM1L* KO cells. **B-C)** Subcellular location of *Salmonella*-secreted SteA or SteA K36A mutant. Representative confocal images (A) and quantification (B) of GFP-LC3^{+} *Salmonella* associated with SteA-V5 or SteA K36A-V5 in WT cells. Cells were infected with the Δ*steA* mutant reconstituted with SteA-V5 or SteA K36A-V5, fixed and imaged at indicated time points post-infection. B) Representative images of infected WT cells at 2 hours post-infection. Insets show magnified (2x) views of the boxed region in each image. Scale bars represent 10μm in full images and 5μm in insets. C) Percentage of GFP-LC3^{+} *Salmonella* with SteA-V5 or SteA K36A-V5 at indicated time points post-infection. Over 500 cells were analyzed. **D)** SteA is required to impair the maturation of *Salmonella*-containing autophagosomes. Percentage of mCherry-LC3^{+} *Salmonella* also positive for GFP in WT and *SACM1L* KO cells at indicated times post-infection. Over 600 cells were analyzed. Data from at least two independent experiments were analyzed using ANOVA (mean ± SEM). **p<0.01, ***p<0.001.
