# Supplementary material for: SAC1 regulates autophagosomal phosphatidylinositol-4-phosphate for xenophagy-directed bacterial clearance
Source: Cell Rep. Author manuscript; Available in PMC 2021 Aug 2. (PMC8327279; doi:10.1016/j.celrep.2021.109434)
Supplement: 4 [file NIHMS1728472-supplement-4.pdf]

Cell Reports

Article

SAC1 regulates autophagosomal phosphatidylinositol-4-phosphate for xenophagy-directed bacterial clearance

Graphical abstract

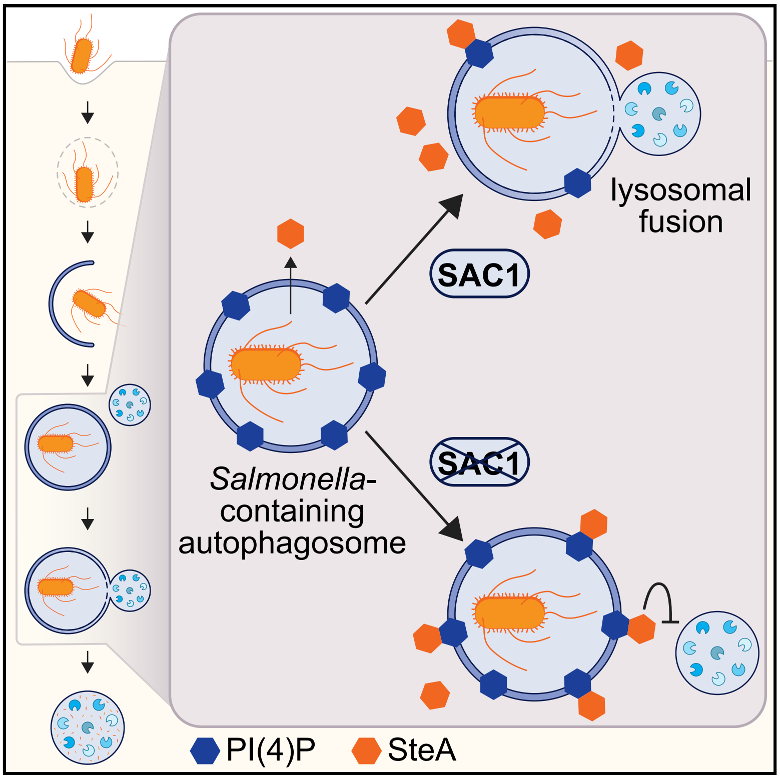

Highlights

- Phosphoinositide kinase and phosphatase screen reveals SAC1 as a xenophagy regulator
- SAC1 deficiency impairs fusion of bacteria-containing autophagosomes with lysosomes
- Loss of SAC1 leads to PI(4)P accumulation on *Salmonella*-containing autophagosomes
- The *Salmonella* effector protein SteA binds excess PI(4)P, impeding lysosomal fusion

Authors

Kai Liu, Lingjia Kong, Daniel B. Graham, Kimberly L. Carey, Ramnik J. Xavier

Correspondence

xavier@molbio.mgh.harvard.edu

In brief

Liu et al. reveal that a mammalian protein regulates levels of its substrate, impairing the function of a bacterial effector during xenophagy. The authors demonstrate that SAC1 restricts intracellular bacterial replication by controlling PI(4)P on *Salmonella*-containing autophagosomes and preventing recruitment of the *Salmonella*-secreted effector SteA, which interferes with lysosomal fusion.

Liu et al., 2021, Cell Reports *36*, 109434
July 27, 2021 © 2021 The Authors.
https://doi.org/10.1016/j.celrep.2021.109434

CellPress

Cell Reports

CellPress
OPEN ACCESS

Article

SAC1 regulates autophagosomal phosphatidylinositol-4-phosphate for xenophagy-directed bacterial clearance

Kai Liu,[1] Lingjia Kong,[1] Daniel B. Graham,[1,2,3,4] Kimberly L. Carey,[2] and Ramnik J. Xavier[1,2,3,4,5,*]

[1]Center for Computational and Integrative Biology, Department of Molecular Biology, Massachusetts General Hospital, Harvard Medical School, Boston, MA 02114, USA

[2]Broad Institute of MIT and Harvard, Cambridge, MA 02142, USA

[3]Center for the Study of Inflammatory Bowel Disease, Massachusetts General Hospital, Harvard Medical School, Boston, MA 02114, USA

[4]Klarman Cell Observatory, Broad Institute of MIT and Harvard, Cambridge, MA 02142, USA

[5]Lead contact

*Correspondence: xavier@molbio.mgh.harvard.edu

https://doi.org/10.1016/j.celrep.2021.109434

SUMMARY

Phosphoinositides are important molecules in lipid signaling, membrane identity, and trafficking that are spatiotemporally controlled by factors from both mammalian cells and intracellular pathogens. Here, using small interfering RNA (siRNA) directed against phosphoinositide kinases and phosphatases, we screen for regulators of the host innate defense response to intracellular bacterial replication. We identify SAC1, a transmembrane phosphoinositide phosphatase, as an essential regulator of xenophagy. Depletion or inactivation of SAC1 compromises fusion between *Salmonella*-containing autophagosomes and lysosomes, leading to increased bacterial replication. Mechanistically, the loss of SAC1 results in aberrant accumulation of phosphatidylinositol-4-phosphate [PI(4)P] on *Salmonella*-containing autophagosomes, thus facilitating recruitment of SteA, a PI(4)P-binding *Salmonella* effector protein, which impedes lysosomal fusion. Replication of *Salmonella* lacking SteA is suppressed by SAC-1-deficient cells, however, demonstrating bacterial adaptation to xenophagy. Our findings uncover a paradigm in which a host protein regulates the level of its substrate and impairs the function of a bacterial effector during xenophagy.

INTRODUCTION

Autophagy is a sequential, highly regulated catabolic process that maintains cellular homeostasis and is further induced in response to internal or external cues (Bento et al., 2016; Clarke and Simon, 2019; Levine and Kroemer, 2008). Evolutionarily conserved autophagy genes coordinate to form a double-membraned autophagosome that engulfs content and fuses with lysosomes for degradation (Levine and Kroemer, 2008). Damaged organelles, protein aggregates, or intracellular pathogens are targeted for degradation through selective autophagy mechanisms (Gatica et al., 2018; Zaffagnini and Martens, 2016). The specificity of selective autophagy in higher eukaryotes is largely conferred by soluble autophagy cargo receptors including SQSTM1, NDP52, TAX1BP1, OPTN, and NBR1 (Gatica et al., 2018; Kirkin and Rogov, 2019). These receptors tether cargo to nascent autophagosomes by simultaneously binding cargo and LC3 family proteins through an LC3-interacting motif (Kim et al., 2016). Cargo is commonly ubiquitinated and bound by receptors through ubiquitin-binding domains (Deosaran et al., 2013; Liu et al., 2012; Mancias et al., 2014; Thurston et al., 2012).

Selective autophagy for the clearance of intracellular pathogens, known as xenophagy, is an important innate defense response, and *Salmonella* serves as a model bacteria susceptible to this defense mechanism (Benjamin et al., 2013; Birmingham et al., 2006; Conway et al., 2013; Huang and Brumell, 2014). Following invasion into a mammalian cell, *Salmonella* reside in *Salmonella*-containing vacuoles (SCVs), which are actively remodeled by bacteria to facilitate replication (Castanheira and García-Del Portillo, 2017; Kehl et al., 2020; Tuli and Sharma, 2019). If SCV membrane integrity is compromised or bacteria escape the vacuole, host autophagy machinery recognizes either damaged SCVs or cytosolic bacteria. Damaged SCVs are detected by galectin family proteins that bind lumenal glycan moieties and mark the membrane for autophagic degradation by direct interaction with cargo receptors (Paz et al., 2010; Thurston et al., 2012). Cytosolic *Salmonella* are ubiquitinated by E3 ligases and bound by cargo receptors by a ubiquitin-binding motif (Heath et al., 2016; Huett et al., 2012; Polajnar et al., 2017; Shaid et al., 2013). Once targeted to intracellular bacteria, NDP52, a xenophagy cargo receptor, initiates autophagy by recruiting the ULK complex, which activates phosphoinositide 3-kinase (PIK3C3) to generate phosphatidylinositol-3-phosphate [PI(3)P] (Ravenhill et al., 2019; Vargas et al., 2019). This recruits effectors, such as WIPI proteins, to autophagosomal membranes (Nakatogawa, 2020; Polson et al., 2010)

This is an open access article under the CC BY license (http://creativecommons.org/licenses/by/4.0/).

followed by LC3 and the lipidation machinery complex that facilitates full encapsulation of bacteria within autophagosomes (Kimmey and Stallings, 2016; Mizushima and Komatsu, 2011). Clearance of autophagosomal content, including LC3 and cargo receptors, follows fusion with lysosomal compartments containing degradative enzymes (Birmingham et al., 2006; Sharma et al., 2018).

As mammalian cells use xenophagy to eliminate intracellular pathogens, bacteria evolved strategies to manipulate or abrogate host cell processes for survival (Gomez-Valero et al., 2019; Huang and Brumell, 2014; Schroeder, 2018; Xiao and Cai, 2020). *Streptococcus pyogenes* expresses a cysteine protease SpeB to evade autophagic recognition by degrading SQSTM1 and NDP52 (Barnett et al., 2013). *Listeria monocytogenes* and *Legionella pneumophila* secrete enzymes targeting LC3 to prevent lipidation and conjugation of autophagosomal membranes (Choy et al., 2012; Horenkamp et al., 2015; Kubori et al., 2017; Mitchell et al., 2018; Tattoli et al., 2013). *Salmonella* secretes effector proteins including SopF, which inhibits the association between ATG16L1 and the vacuolar ATPase component ATP6V0C, thus blocking ATG16L1 and LC3 recruitment and xenophagy initiation (Lau et al., 2019; Mesquita et al., 2012; Xu et al., 2019).

Pathogens also exploit membrane trafficking to establish and maintain replication-competent niches in the host cytosol. Phosphoinositides are key components of cellular membranes essential for spatiotemporal regulation of trafficking. Seven phosphorylated forms of phosphoinositide are critical for cellular physiology and membrane identity, and interconversion between these forms is tightly controlled by lipid kinases and phosphatases (Dall'Armi et al., 2013; Nakada-Tsukui et al., 2019). Phosphatidylinositol-4-phosphate [PI(4)P], found on Golgi, endosomes, and plasma membranes, has important signaling roles in trafficking (Santiago-Tirado and Bretscher, 2011), phagolysosome resolution (Levin-Konigsberg et al., 2019), inflammasome formation (Chen and Chen, 2018), and autophagy (de la Ballina et al., 2020; De Tito et al., 2020; Miao et al., 2020; Wang et al., 2015; Yamashita et al., 2006). In humans, PI(4)P homeostasis is coordinately maintained by four membrane-associated phosphatidylinositol-4 kinases (PI4Ks) and a single conserved PI(4)P phosphatase, SAC1 (Clayton et al., 2013; Del Bel and Brill, 2018; Liu et al., 2009; Venditti et al., 2019; Wang et al., 2013). Interestingly, *L. pneumophila* secretes lipid kinases (LepB) and phosphatases (SidC and SidF) capable of regulating PI(4)P levels on *Legionella*-containing vacuolar membranes as well as phosphoinositide-binding proteins that localize to *Legionella*-containing vacuoles (Hubber et al., 2014; Luo et al., 2015; Nachmias et al., 2019; Wasilko and Mao, 2016; Weber et al., 2018).

The role of lipid membrane composition in selective autophagy, however, is largely unknown. In this study, we completed a targeted small interfering RNA (siRNA) screen to find lipid kinases and phosphatases functioning in bacterial autophagy. We identified the PI(4)P phosphatase SAC1, encoded by human *SACM1L*, as an essential regulator of *Salmonella*-induced xenophagy. Our data demonstrate that the control of PI(4)P levels on autophagosomal membranes by SAC1 is required for efficient intracellular bacterial defense. We found that elevated PI(4)P levels on *Salmonella*-containing autophagosomes in *SACM1L*-deficient cells delays fusion with lysosomes. Furthermore, we showed that the *Salmonella* type III secreted effector and PI(4)P-binding protein SteA promotes intracellular bacterial replication by impeding clearance through xenophagy. Collectively, our results reveal counter-regulation of lipid membrane dynamics by mammalian host cells and bacteria during xenophagy to modulate an innate defense response.

RESULTS

Lipid enzymes function in intracellular bacterial replication

We performed a directed siRNA screen to investigate the specific roles of membrane phosphoinositides in host bacterial defense. We targeted 67 known human lipid kinase and phosphatase genes by using three independent siRNA molecules per gene and evaluated transfected HeLa cells for changes in replication of bioluminescent *Salmonella enterica* serovar Typhimurium at 8.5 h post-infection relative to non-targeting siRNA controls (Figure 1A; Table S1; Dickson and Hille, 2019; Sacco et al., 2012; Sasaki et al., 2009). Knockdown of *PIK3C3*, which is required to generate PI(3)P for nucleation of the autophagosomal membrane (Simonsen and Tooze, 2009; Yue and Zhong, 2010), increased intracellular bacterial replication compared to controls, as expected, and served as a positive control (Figure 1A). *SACM1L* was the only additional gene in our screen required to restrict intracellular bacterial replication.

We also identified phosphoinositide regulators that supported intracellular replication including MTMR4, a PI(3)P phosphatase required for vesicular trafficking and maturation of endocytic and autophagic compartments (Pham et al., 2018; Figure 1A). In agreement with our data, previous reports demonstrated that expression of MTMR4 supports *Salmonella* replication by maintaining PI(3)P levels and stabilizing SCVs (Teo et al., 2016). Several other genes identified have no known activity in bacterial defense (*TMEM55A*, *TMEM55B*, *PLPPR4*, and *PI4K2α*). TMEM55A and TMEM55B are PI(4,5)P2 phosphatases that localize to late endosomal and lysosomal membranes (Morioka et al., 2018). Previous studies revealed that TMEM55B is controlled by TFEB, a master regulator of lysosomal biogenesis, and functions in lysosomal positioning (Hashimoto et al., 2018; Takemasu et al., 2019; Willett et al., 2017). Production of PI(4)P by PI4K2α, one of four human PI4Ks, has been implicated in autophagosome-lysosomal fusion (Albanesi et al., 2015; Wang et al., 2015). Knockdowns of the other human PI4Ks (*PI4Kα*, *PI4Kβ*, and *PI4K2β*) did not significantly modulate *Salmonella* replication (Table S1).

Of the lipid kinases and phosphatases screened, PI4K2α and SAC1 were the only enzymes found that catalyze opposing reactions on the same substrate. As *SACM1L* is evolutionarily conserved across eukaryotic cells and the only PI(4)P-specific phosphatase in humans (Zhang et al., 2020), we focused our investigations on its role in restricting intracellular bacterial replication. To maintain a synchronous infection, the window for infections throughout our study was limited to 30 min (unless otherwise stated), after which cells were immediately analyzed or washed and treated with gentamicin to prevent further infection by the remaining extracellular bacteria. Time post-infection

CellPress
OPEN ACCESS

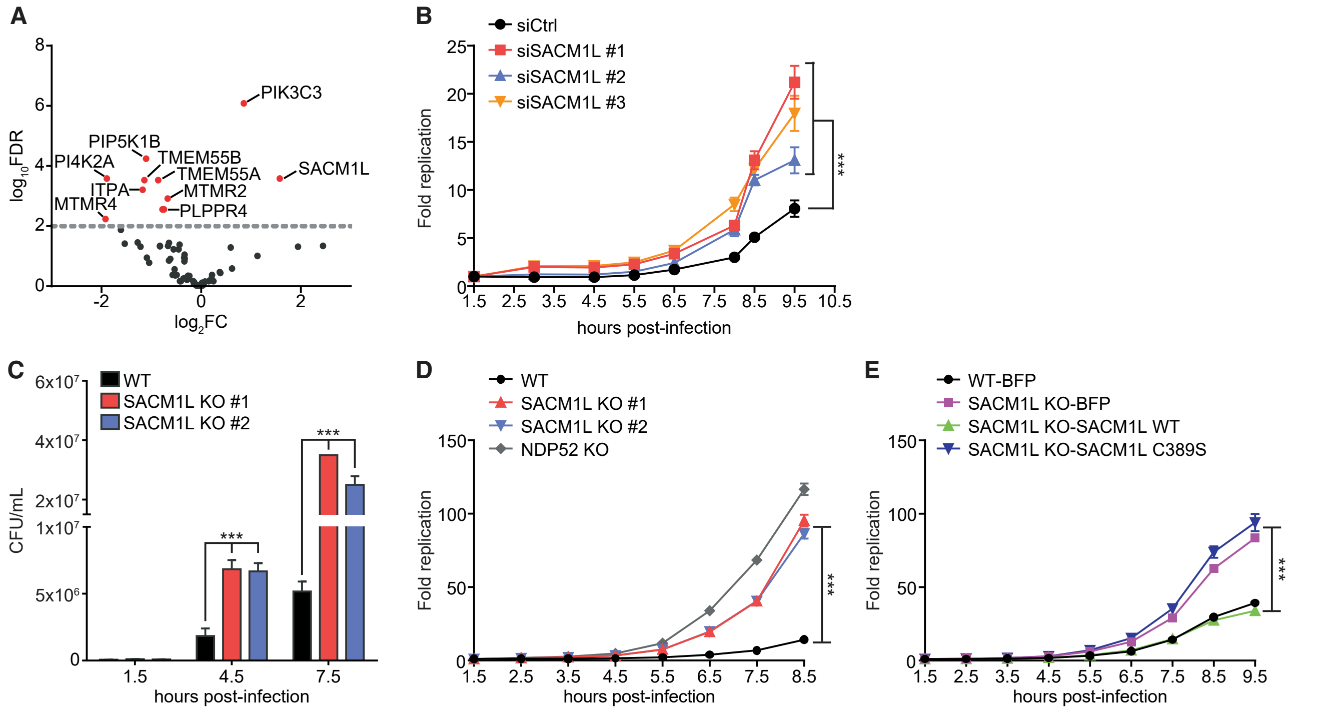

Figure 1. SAC1 restricts intracellular bacterial replication

(A) Volcano plot of siRNA screen shows $\log_2$ fold change ($\log_2$FC) of *Salmonella* replication compared with that of control siRNA. Data represent combined analysis from three independent experiments. Red dots indicate genes with false discovery rate (FDR) values of <0.01.

(B) HeLa cells transfected with control (Ctrl) or one of three independent *SACM1L*-directed siRNA molecules were infected with *Salmonella* expressing bacterial luciferase. Luciferase levels were measured over time. Bacterial replication was normalized to baseline infection at 1.5 h post-infection.

(C) CFU/mL of *Salmonella* at indicated times after infection of WT or *SACM1L* KO cells.

(D) Fold change of luciferase-expressing *Salmonella* replication in WT, *SACM1L* KO, and *NDP52* KO cells

(E) WT cells stably expressing BFP and *SACM1L* KO cells stably expressing BFP, *SACM1L* WT, or *SACM1L* C389S were infected with luciferase-expressing *Salmonella*. Luciferase levels were measured over time. Bacterial replication was normalized to baseline infection at 1.5 h post-infection. For all quantifications, three independent experiments were analyzed using ANOVA (mean ± SEM [standard error of the mean]). ***$p < 0.001$.

See also Figure S1 and Tables S1 and S2.

designates the time elapsed from the beginning of the infection period. We first confirmed the knockdown phenotype in an independent experiment by using three *SACM1L* targeting and non-targeting control siRNA molecules. Cells transfected with each of the individual *SACM1L*-targeting siRNA molecules were defective in restricting *Salmonella* replication as compared to control cells (Figure 1B).

SAC1 phosphatase activity is required for restricting bacterial replication

To validate our siRNA knockdown results, we generated *SACM1L* knockout (KO) HeLa cell lines by using the CRISPR-Cas9 system. Two independent *SACM1L* KO clones showed no detectable SAC1 expression by immunoblot and immunofluorescence microscopy and exhibited dispersion of the trans-Golgi network as previously reported (Liu et al., 2008; Figures S1A and S1B). With these *SACM1L* KO clones, we performed two independent *Salmonella* replication assays. Wild-type (WT) cells infected with *S.* Typhimurium SL1344 or an SL1344 strain expressing luciferase restricted intracellular replication as measured by colony-forming units (CFUs) and bioluminescence intensity, respectively (Figures 1C and 1D; Conway et al., 2013; Hoiseth and Stocker, 1981). Similar to *NDP52* KO cells, robust bacterial replication was observed in cells lacking SAC1, confirming *SACM1L* KO cells reproduce the *SACM1L* knockdown phenotype (Figures 1C and 1D).

To verify that disruption to *SACM1L* specifically was responsible for the defect in bacterial replication restriction and to control for off-target effects, we re-expressed the SAC1 WT protein or blue fluorescent protein (BFP) control in *SACM1L* KO cells. Expression of SAC1 WT in *SACM1L* KO and WT cell lines was confirmed by immunoblot (Figure S1A). *SACM1L* KO cells reconstituted with SAC1 WT restricted bacterial replication to a level similar to that of WT cells expressing BFP (Figure 1E). These data establish that the loss of SAC1 is responsible for increased bacterial replication. Furthermore, to determine if SAC1 phosphatase activity was required for bacterial growth restriction, we substituted the catalytic cysteine residue with serine and reconstituted *SACM1L* KO cells with the catalytically dead SAC1 C389S mutant (Liu et al., 2009). SAC1 C389S expression was confirmed by immunoblot and comparable to SAC1 WT levels (Figure S1A). Expression of SAC1 C389S did not rescue the

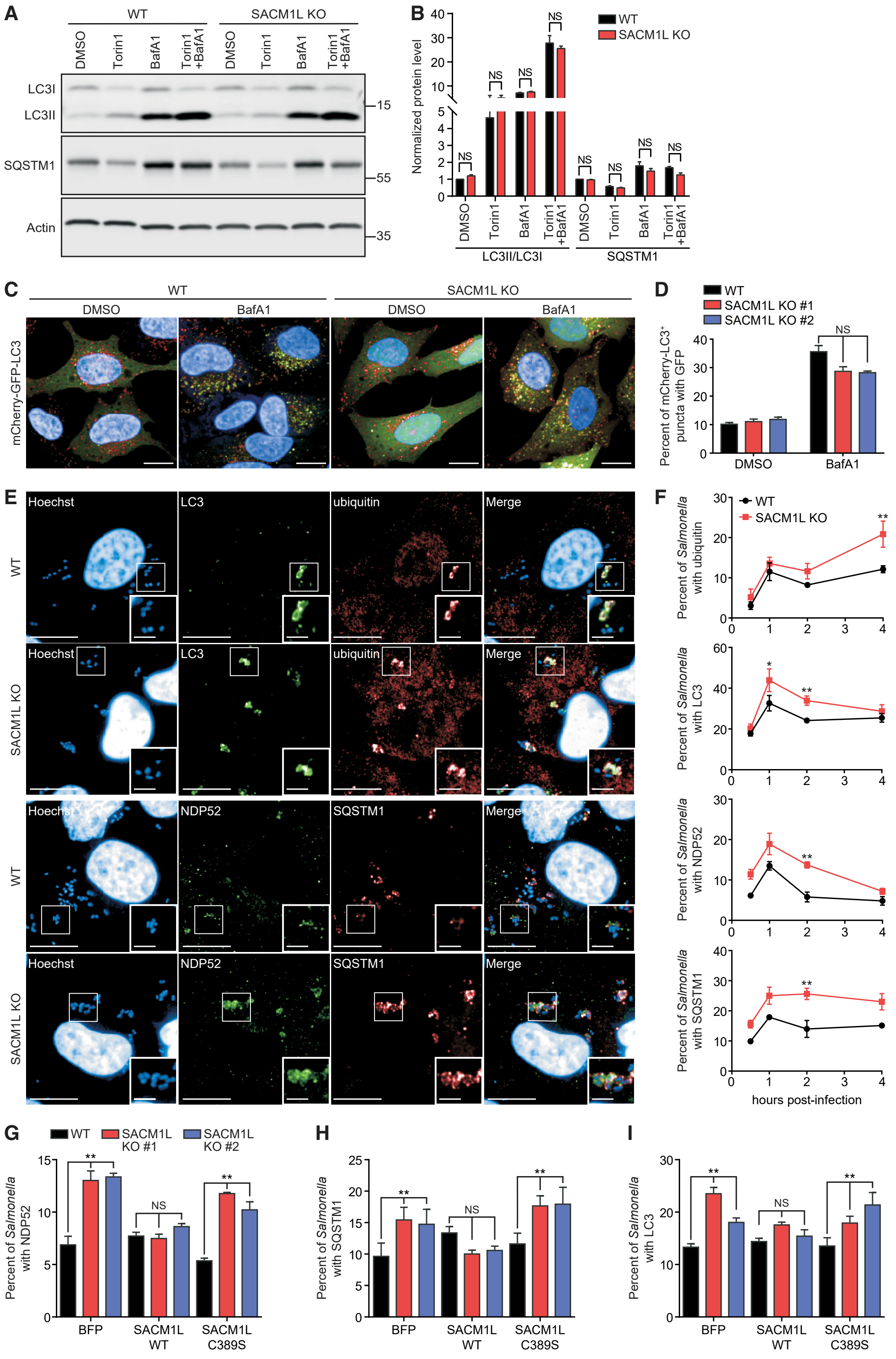

(legend on next page)

CellPress
OPEN ACCESS

SACM1L KO phenotype, indicating SAC1 phosphatase activity is required for restricting intracellular bacterial replication (Figure 1E).

SAC1 does not affect basal or non-selective autophagic flux nor lysosomal function

Next, we interrogated the role of SAC1 in innate defense mechanisms used to restrict bacterial replication by examining the effect of SAC1 expression on autophagy and autophagic flux. The ratio of membrane-bound LC3II to cytosolic LC3I, which corresponds to autophagosome formation, was consistent between WT and *SACM1L* KO cells under basal conditions as well as in response to treatment with the autophagy-inducing small molecule Torin1 or lysosomal inhibitor Bafilomycin A1 (BafA1; Figures 2A and 2B). Additionally, no difference was detected in the LC3I to LC3II conversion in WT, *SACM1L* KO, or reconstituted cells at steady state or in response to amino acid starvation (Figures S2A and S2B). SQSTM1 protein levels in both WT and *SACM1L* KO cells decreased in response to Torin1 and increased upon treatment with BafA1 alone or in combination with Torin1, indicating that the loss of SAC1 did not alter basal or non-selective autophagy (Figures 2A and 2B).

We then generated WT and *SACM1L* KO cells that stably express a tandem mCherry-GFP-LC3 reporter to determine if SAC1 alters autophagosome maturation. Because GFP fluorescence is quenched in acidic lysosomal compartments, $mCherry^{+}GFP^{+}$ vesicles represent immature autophagosomes, whereas $mCherry^{+}GFP^{-}$ vesicles reveal acidified autolysosomes (Hansen and Johansen, 2011). By quantitative microscopy, we found that the percentage of immature autophagosomes and autolysosomes did not change significantly in *SACM1L* KO compared to WT cells treated with DMSO or BafA1 (Figures 2C and 2D). Lysosomal number, indicated by the intensity of pH-sensitive LysoTracker dye, and function, measured by hydrolyzed DQ-green BSA, were also unchanged in *SACM1L* KO cells (Figures S2C and S2D). These results indicate that SAC1 loss does not alter steady-state or induced non-selective autophagic flux, autophagosome maturation, or lysosomal function.

SAC1 phosphatase activity is required for functional xenophagy

Considering that SAC1 loss did not interfere with non-selective autophagy or lysosomal function, we investigated if SAC1 restricts intracellular bacterial replication through a xenophagy-specific role. We examined the effect of SAC1 expression on bacterial targeting by key xenophagy markers by using time-dependent quantitative confocal imaging. As intracellular bacteria are recognized, host xenophagy machinery, including ubiquitin, cargo receptors (NDP52 and SQSTM1), galectins (Gal3), and isolation membranes (marked by LC3), are recruited and associate with bacteria by 1 h post-infection (Paz et al., 2010; Thurston et al., 2012; Xu et al., 2019). By 2 h post-infection, xenophagy markers are degraded by lysosomal fusion, and detectable associations with bacteria decline to steady-state levels. A higher percentage of *Salmonella* was associated with endogenous LC3, NDP52, and SQSTM1 at 2 h post-infection in *SACM1L* KO cells than in WT cells, suggesting a delay in *Salmonella*-containing autophagosome maturation (Figures 2E and 2F). Consistent with these data, we observed a slower rate of NDP52 degradation after *Salmonella* infection in *SACM1L* KO by immunoblot analysis (Figures S2E and S2F). Although levels of SQSTM1 associated with bacteria were elevated, no delay in total cellular SQSTM1 turnover was detected (Figures S2E and S2G). In contrast, loss of SAC1 did not alter the percentage of bacteria that co-localized with endogenous ubiquitin, LC3, NDP52, or SQSTM1 at earlier times (0.5 h and 1 h post-infection), indicating that SAC1 does not function in the recognition or assembly of autophagy machinery around intracellular *Salmonella* (Figure 2F).

Changes in membrane composition by knockdown of OSBP1, a PI(4)P- and cholesterol-binding protein that interacts with *Salmonella* effectors SseJ and SseL for recruitment to the SCV, were reported to destabilize SCV membranes (Kolodziejek et al., 2019). We monitored the dynamic recruitment of GFP-tagged Gal3 to intracellular dsRed-expressing *Salmonella* (Maejima et al., 2013; Rioux et al., 2007) but did not detect differences in co-localization in WT and *SACM1L* KO cells, suggesting that the loss of SAC1 does not affect SCV integrity at 1 h post-infection (Figures S2H and S2I). These data, in addition to the observed defect in the restriction of bacterial replication (Figure 1), suggest that SAC1 loss delays the maturation of autophagosomes and degradation of *Salmonella* targeted by xenophagy cargo receptors. Furthermore, re-expression of SAC1 WT, but not SAC1 C389S or BFP, reduced the levels of $NDP52^{+}$, $SQSTM1^{+}$, and $LC3^{+}$ *Salmonella* in *SACM1L* KO cells at 2 h post-infection (Figures 2G–2I), revealing that SAC1 phosphatase activity is necessary for functional xenophagy.

To determine if SAC1 functions in other types of selective autophagy, we induced mitophagy or aggrephagy in WT and

Figure 2. SAC1 activity regulates maturation of *Salmonella*-containing autophagosomes

(A and B) Representative immunoblot (A) and quantification (B) of LC3 conversion and SQSTM1 in WT and *SACM1L* KO cells treated with 0.1% DMSO, 1 μM Torin1, 200 ng/ml BafA1, or a combination of Torin1 and BafA1 for 4 h. Quantification of LC3II/LC3I ratio or SQSTM1 protein was normalized to DMSO-treated WT cells.

(C and D) Representative confocal images (C) and quantification (D) of mCherry-GFP-LC3 expression in WT and *SACM1L* KO cells treated with DMSO or BafA1 for 4 h. Scale bars represent 20 μm.

(E and F) Representative confocal images (E) and quantifications (F) of *Salmonella* associated with endogenous ubiquitin, LC3, NDP52, and SQSTM1 in WT and *SACM1L* KO cells at indicated times post-infection. Images were captured at 2 h post-infection. Insets are boxed regions magnified (1.8×). Hoechst shows HeLa cell nuclei and *Salmonella*. Scale bars represent 20 μm in full images and 5 μm in insets.

(G–I) Percentage of *Salmonella* associated with endogenous NDP52 (G), SQSTM1 (H), or LC3 (I) in WT and *SACM1L* KO cells stably expressing BFP, *SACM1L* WT, or *SACM1L* C389S at 2 h post-infection. For all quantifications, over 500 cells were analyzed. Three independent experiments were analyzed using ANOVA (mean ± SEM). *$p < 0.05$, **$p < 0.01$; NS, not significant.

See also Figure S2.

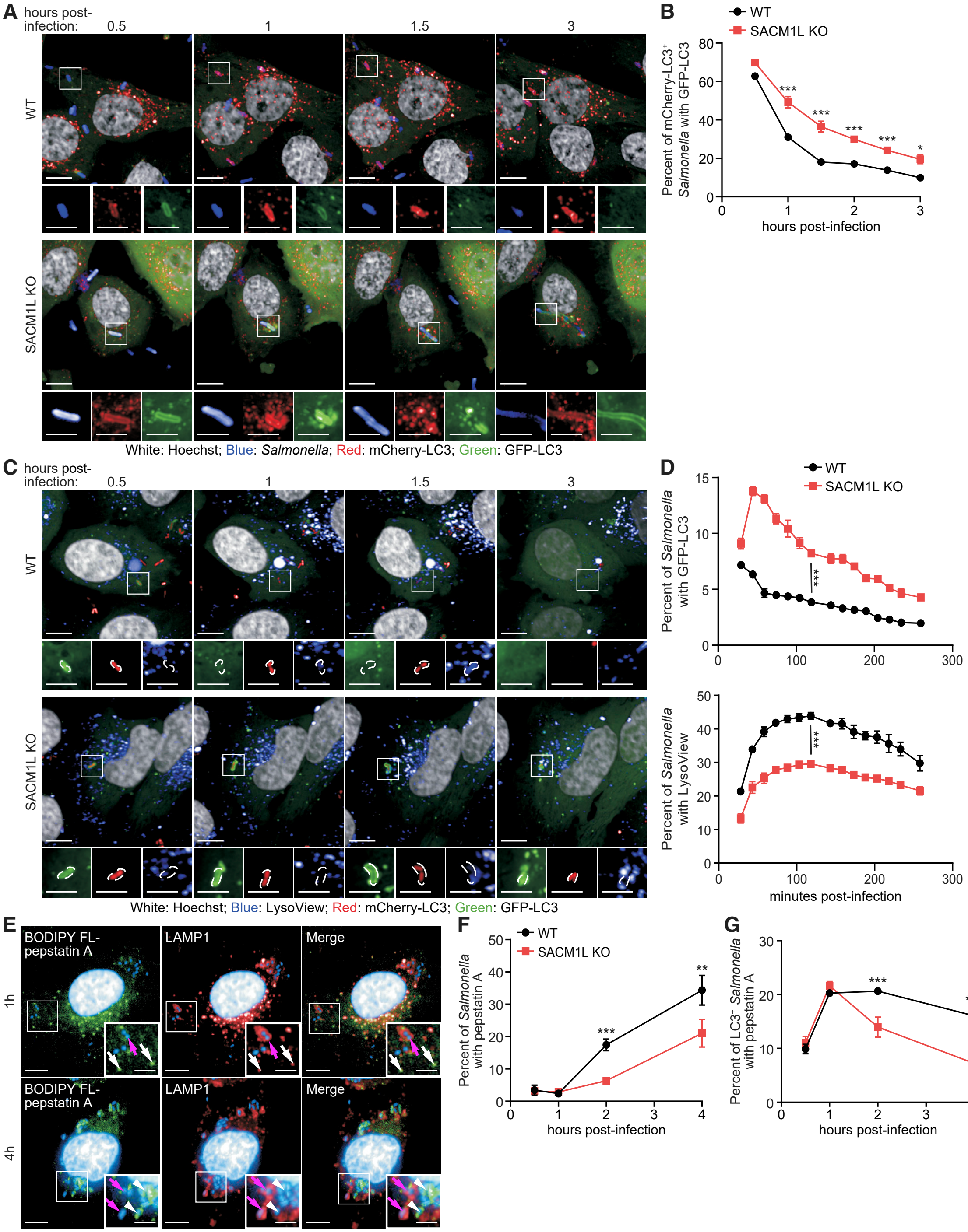

Figure 3. SAC1 loss impairs lysosomal fusion

(A and B) WT and *SACM1L* KO cells stably expressing mCherry-GFP-LC3 were stained with Hoechst, infected with *Salmonella* labeled with CellTracker deep red dye, washed, imaged by live confocal microscopy (A), and quantified (B). Percentage of GFP-LC3$^+$ *Salmonella*-containing autophagosomes is shown at indicated times post-infection.

(C and D) WT and *SACM1L* KO cells stably expressing GFP-LC3 were infected with dsRed-expressing *Salmonella* and stained with LysoView 633 dye and Hoechst. Live confocal microscopy images (C) and percentage (D) of *Salmonella* positive for GFP-LC3 (top) or LysoView dye (bottom) are shown. Magnified images (2.4×) show separated channels of the boxed region in merged images. Scale bars represent 10 μm in merged images and 5 μm in magnified images.

(legend continued on next page)

SACM1L KO cells. Similar to xenophagy, Parkin-mediated mitophagy uses NDP52 as a cargo receptor to clear damaged mitochondria (Heo et al., 2015; Lazarou et al., 2015). WT and *SACM1L* KO cells expressing Parkin were treated with either carbonyl cyanide 3-chlorophenylhydrazone (CCCP) or a combination of oligomycin and antimycin A to induce mitochondrial depolarization (Yamano et al., 2014). As detected by TOMM20, a mitochondrial outer membrane protein, both WT and *SACM1L* KO cells efficiently cleared damaged mitochondria (Figures S2J and S2K). Similarly, *SACM1L* WT and KO cells treated for 2 h with puromycin, an amino acid analog that induces protein aggregates by prematurely terminating translation, effectively cleared ubiquitin$^+$ aggregates after 3-h and 5-h periods of recovery (Figures S2L–S2N). As previously shown in response to a loss of other autophagy cargo receptors, *SACM1L* KO cells formed fewer and smaller puromycin-induced aggregates (Bjørkøy et al., 2005; Sarraf et al., 2020), but there was no effect on the rate of clearance (Figures S2O and S2P). These results indicate that SAC1 is not required for Parkin-mediated mitophagy or aggrephagy but rather functions specifically in xenophagy.

SAC1 promotes the maturation of *Salmonella*-containing autophagosomes

Next, we examined which step in xenophagy is modulated by SAC1. Using the WT and *SACM1L* KO mCherry-GFP-LC3 reporter cell lines described above, we monitored the maturation of *Salmonella*-containing autophagosomes by live cell imaging. CellTracker-labeled *Salmonella* were detected first in mCherry$^+$GFP$^+$ autophagosomes, which gradually converted to mCherry$^+$GFP$^-$ autolysosomes. In WT cells, only 17% of *Salmonella*-containing autophagosomes were immature at 2 h post-infection (Figures 3A and 3B). In contrast, 30% of *Salmonella* remained in mCherry$^+$GFP$^+$ autophagosomes in *SACM1L* KO cells, suggesting that the loss of SAC1 delays maturation of *Salmonella*-containing autophagosomes (Figures 3A and 3B).

To determine if this defect was due to SAC1 loss impeding the closure of autophagosomal membranes, we monitored the recruitment and removal of endogenous WIPI2 on LC3$^+$ *Salmonella*. WIPI2 functions in conjunction with LC3 to expand isolation membranes and dissociates from LC3$^+$ membranes prior to autophagosome closure (Dooley et al., 2014; Fracchiolla et al., 2020). In both WT and *SACM1L* KO cells, WIPI2 was efficiently recruited to LC3$^+$ *Salmonella* and then gradually disappeared, as demonstrated by the decreasing percentage of WIPI2$^+$LC3$^+$ among all LC3$^+$ *Salmonella* over time (Figures S3A and S3B). These data indicate that SAC1 is not required for the formation and closure of autophagosomes around *Salmonella*.

To directly assess the fusion of acidic compartments with *Salmonella*-containing autophagosomes, we simultaneously monitored *Salmonella*, GFP-LC3, and lysosomes stained with a pH-sensitive LysoView dye. In WT cells, GFP-LC3 co-localized with *Salmonella* at 30 min post-infection, and the loss of detectable GFP-LC3$^+$ *Salmonella* was coordinated with the increase of LysoView dye associated with bacteria, reflecting degradation of GFP-LC3 upon fusion with lysosomes (Figures 3C and 3D). In *SACM1L* KO cells, a higher percentage of *Salmonella* was associated with GFP-LC3 at all times (Figure 3D). Furthermore, only 30% of *Salmonella* was localized to LysoView$^+$ acidic compartments in *SACM1L* KO cells at 120 min post-infection as compared to 44% in WT cells, confirming that SAC1 loss delays the fusion of *Salmonella*-containing autophagosomes with lysosomes (Figures 3D).

We then used BODIPY FL-pepstatin A, which selectively binds to active cathepsin D within lysosomes to detect the delivery of lysosomal enzymes to *Salmonella*-containing autophagosomes (Chen et al., 2000). As expected, most *Salmonella* bacteria in WT cells were found residing within LAMP1$^+$pepstatin A$^-$ vacuoles at 1 h post-infection (Figures 3E and 3F; Birmingham et al., 2006(Zoncu et al., 2011)). By 2 h post-infection, *Salmonella* targeted by xenophagy machinery in WT cells progressed to LAMP1$^-$pepstatin A$^+$ compartments, indicating that active lysosomes fused with *Salmonella*-containing autophagosomes (Figure 3F). In contrast, the loss of SAC1 reduced the delivery of lysosomal enzymes to *Salmonella*-containing autophagosomes, as reflected by the lower percentage of bacteria with pepstatin A (Figure 3F) as well as the lower percentage of LC3$^+$pepstatin A$^+$ *Salmonella* in *SACM1L* KO cells (14%) than in WT cells (21%) by 2 h post-infection (Figure 3G). This result was further validated using a MagicRed assay, which reflects cathepsin B activity, and DQ-BSA, which indicates the cleavage capacity of lysosomal hydrolases. The percentages of both MagicRed$^+$ and DQ-BSA$^+$ *Salmonella* by 2 h post-infection were lower in *SACM1L* KO cells than in WT cells (Figures S3C–S3F). Collectively, these results indicate that SAC1 promotes fusion of *Salmonella*-containing autophagosomes with lysosomes.

SAC1-dependent maturation of *Salmonella*-containing autophagosomes reduces cytosolic bacterial replication

Not all *Salmonella* bacteria are captured by autophagosomes in epithelial cells; some replicate within SCVs or escape SCVs and replicate within the host cytosol (Castanheira and García-Del Portillo, 2017). To evaluate *Salmonella* existing within these compartments, we monitored WT and *SACM1L* KO cells co-expressing GFP-LC3 (marking autophagosomes) and LAMP1-mCherry (marking SCVs) by live cell imaging for 6 h post-infection (Lane et al., 2019; Valdivia and Falkow, 1996). In WT cells, we observed instances in which a reduction in LAMP1 signal detected on bacteria corresponded with an increase in LC3 signal (Figure 4A). Subsequently, the LC3 signal diminished and the LAMP1 signal

(E) Co-immunostaining of BODIPY FL-pepstatin A, LAMP1, and Hoechst in WT cells at 1 h and 4 h post-infection. Insets are boxed regions magnified (1.8×). Scale bars represent 10 μm in full images and 5 μm in insets. Magenta arrows indicate LAMP1$^+$pepstatin A$^-$ *Salmonella*. White arrows (1-h image insets) indicate pepstatin A$^+$ lysosomes. White arrowheads (4-h image insets) indicate pepstatin A$^+$ *Salmonella*.
(F and G) Percentage of *Salmonella* (F) or LC3$^+$ *Salmonella* (G) associated with pepstatin A in WT and *SACM1L* KO cells at indicated times post-infection. For all quantifications, over 500 cells were analyzed for each condition. Three independent experiments were analyzed using ANOVA (mean ± SEM). $^*p < 0.05$, $^{**}p < 0.01$, $^{***}p < 0.001$.
See also Figure S3.

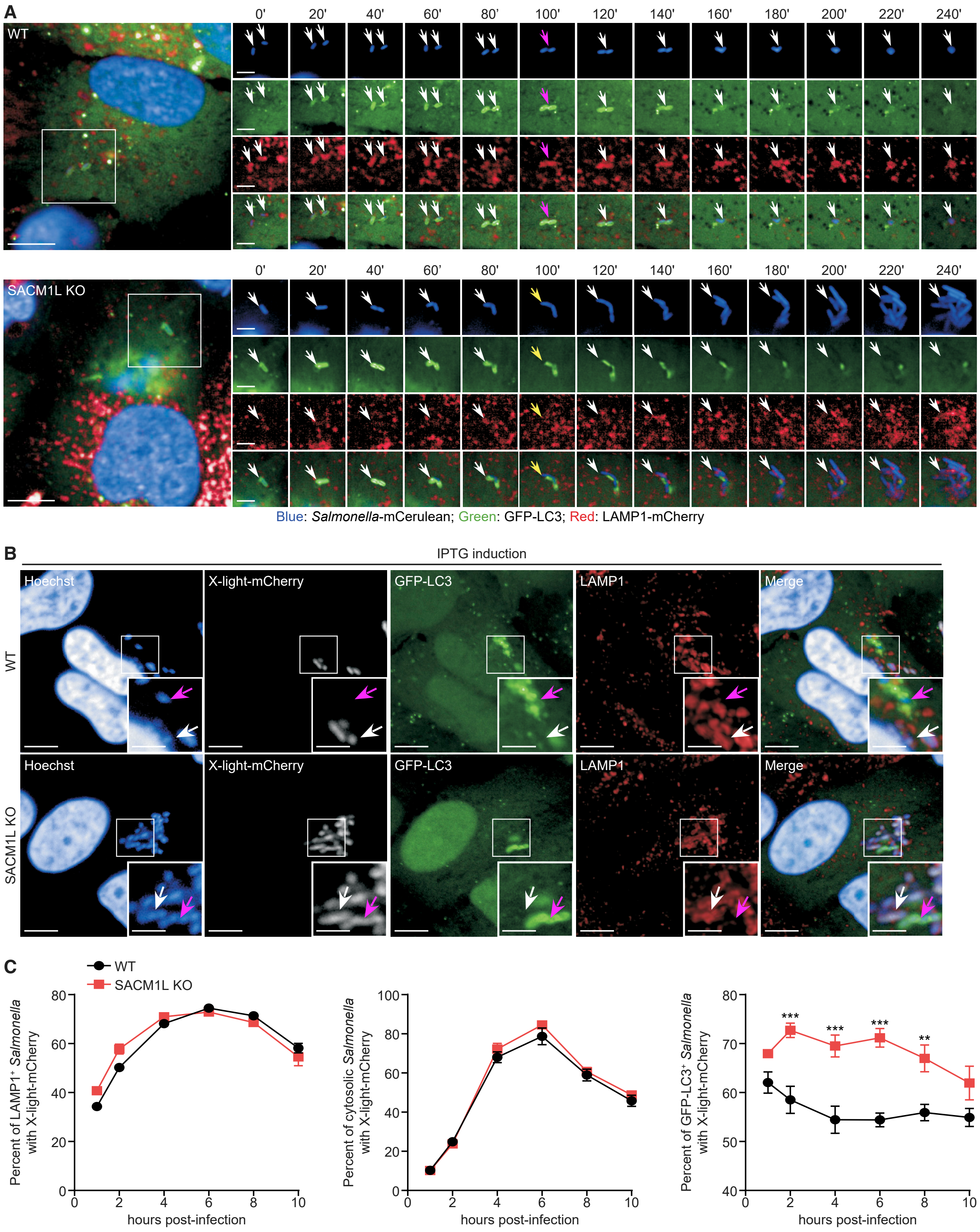

(legend on next page)

increased as the bacterial morphology condensed. These results suggest that bacteria can escape LAMP1+ SCVs and be targeted and degraded through xenophagy. In *SACM1L* KO cells, we observed bacteria that were targeted by autophagy machinery, as detected by GFP-LC3, but did not accumulate LAMP1 (Figure 4A). These bacteria then began to lose detectable GFP-LC3 and rapidly divide in the host cytosol. Similar bacterial populations were not observed in WT cells, suggesting that a delay in *Salmonella*-containing autophagosome maturation due to SAC1 loss may facilitate bacterial escape and replication in the cytosol and contribute to the increased replication phenotype. We cannot, however, exclude the possibility that SAC1 loss also affects SCV stability, leading to bacterial escape.

In a separate experiment, we quantitated LC3+, LAMP1+, and cytosolic LC3−LAMP1− bacteria in WT and *SACM1L* KO cells (Figure S4A). The percentage of cytosolic *Salmonella* increased and the percentage of LAMP1+ *Salmonella* decreased in *SACM1L* KO cells as compared to WT cells, supporting our live imaging observations. Additionally, the percentage of LC3+ *Salmonella* was higher in *SACM1L* KO cells than in WT cells at 2 h post-infection, which is indicative of a delay in autophagosomal maturation and LC3 turnover.

Next, we sought to determine the effect of SAC1 expression on the metabolic activity of *Salmonella* in autophagosomes, SCVs, and the host cytosol. We generated a *Salmonella* strain expressing an isopropyl β-D-1-thiogalactopyranoside (IPTG)-inducible mCherry plasmid (x-light-mCherry) as a reporter of metabolic activity (Sirianni et al., 2016). In the absence of IPTG induction, x-light-mCherry was not expressed by *Salmonella* (Figure S4B). When IPTG was added 30 min prior to fixation, the x-light-mCherry signal was detected in LC3+, LAMP1+, and cytosolic LC3−LAMP1− *Salmonella* (Figure 4B). Quantification of mCherry+LAMP1+ and mCherry+LC3−LAMP1− bacterial populations in WT and *SACM1L* KO cells indicated that the metabolic activity and survival of *Salmonella* in SCVs and the host cytosol are independent of SAC1 (Figure 4C). Consistent with our previous data, we detected a higher percentage of metabolically active *Salmonella* within LC3+ autophagosomes in *SACM1L* KO cells than in WT cells (Figure 4C), indicating a delay in bacterial killing.

To further support these findings, we treated infected WT and *SACM1L* KO cells with chloroquine, which accumulates and kills bacteria in SCVs (Knodler et al., 2014). In the absence of chloroquine, we observed robust *Salmonella* replication in *SACM1L* KO cells compared with that in WT cells (Figure S4C). Chloroquine treatment reduced the number of replicating bacteria in both WT and *SACM1L* KO cells but did not abolish the SAC1-dependent increase in bacterial replication. In addition to supporting a role for SAC1 in autophagosome maturation, these results suggest that the function of SAC1 in lysosomal fusion may impede *Salmonella* escape from immature autophagosomes.

SAC1 loss leads to excessive PI(4)P accumulation on *Salmonella*-containing autophagosomes

We next investigated how the known SAC1 substrate PI(4)P is regulated during xenophagy. We measured PI(4)P levels in WT and *SACM1L* KO cells at steady state and following infection. To specifically detect PI(4)P on subcellular membranes and organelles, we used a previously described fixation protocol that reduces the detection of PI(4)P on the plasma membrane (Hammond et al., 2009). As expected, SAC1 loss elevated PI(4)P levels in uninfected and infected cells (Figures 5A and 5B).

PI(4)P has been observed on starvation-induced, non-selective autophagosomes (Miao et al., 2020; Wang et al., 2015). To investigate if PI(4)P is present on *Salmonella*-containing autophagosomes and SCVs, we detected PI(4)P on LC3+ and LAMP1+ *Salmonella* in WT and *SACM1L* KO cells by immunofluorescence confocal microscopy. Loss of SAC1 increased the percentage of LC3+ autophagosomes associated with PI(4)P as well as PI(4)P fluorescence intensity (Figures 5C, 5D, and S5A). Although endogenous PI(4)P was detectable on less than 5% of *Salmonella*-containing autophagosomes in WT cells, the percentage doubled in *SACM1L* KO cells (Figure 5D). In contrast, we did not detect an increase in the percentage of LAMP1+ SCVs associated with PI(4)P or in PI(4)P fluorescence intensity on SCVs (Figures 5C, 5E, and S5A).

To better understand how PI(4)P is regulated by SAC1 during infection, we tracked its dynamics by live cell imaging using a PI(4)P-specific probe called BFP-2xP4M. The percentage of *Salmonella*-containing autophagosomes with detectable PI(4)P levels (i.e., GFP-LC3+BFP-2xP4M+ *Salmonella*) in *SACM1L* KO cells was initially similar to WT cells, which remained below 5% but reached a maximum of 12% by 150 min post-infection (Figures 5F and 5G), demonstrating that PI(4)P accumulates on *Salmonella*-containing autophagosomes upon SAC1 loss.

To test whether excessive PI(4)P on autophagosomal membranes impairs xenophagy in SAC1-deficient cells, we assessed the combined effect of PI4K knockdown in *SACM1L* KO cells on bacterial targeting and replication. Of the four human PI4Ks, only knockdown of *PI4K2α* reduced *Salmonella* replication in WT cells

Figure 4. SAC1 loss impairs the ability of autophagosomes to restrict *Salmonella* replication

(A) WT and *SACM1L* KO cells stably transduced with GFP-LC3 and LAMP1-mCherry were stained with Hoechst for 10 min, infected with mCerulean-expressing *Salmonella* for 15 min, washed, and imaged by live confocal microscopy every 20 min for 6 h. Timescale 0' to 240' is the minutes from detection of the bacteria in the cell and focal plane. White arrows indicate *Salmonella*. Magenta arrows (top) indicate peak GFP-LC3 intensity. Yellow arrows (bottom) show *Salmonella* escaping from autophagosomes prior to replicating in the host cytoplasm. Image series are boxed regions magnified (1.8×). Scale bars represent 10 μm in image series and 5 μm in full images.

(B) Representative confocal images of IPTG-induced mCherry expression in *Salmonella* within LAMP1+ (SCV) or GFP-LC3+ (autophagosome) compartments in WT and *SACM1L* KO cells at 6 h post-infection. Magenta arrows indicate GFP-LC3+ *Salmonella*. White arrows indicate LAMP1+ *Salmonella*. Insets are boxed regions magnified (2×). Scale bars represent 10 μm in full images and 5 μm in insets.

(C) Percentage of induced mCherry signal in LAMP1+, GFP-LC3−LAMP1−, (cytosolic), or GFP-LC3+ *Salmonella* in WT and *SACM1L* KO cells. For quantification, over 3,000 bacteria were analyzed. Two independent experiments were analyzed using ANOVA (mean ± SEM). $^{**}p < 0.01$, $^{***}p < 0.001$.

See also Figure S4.

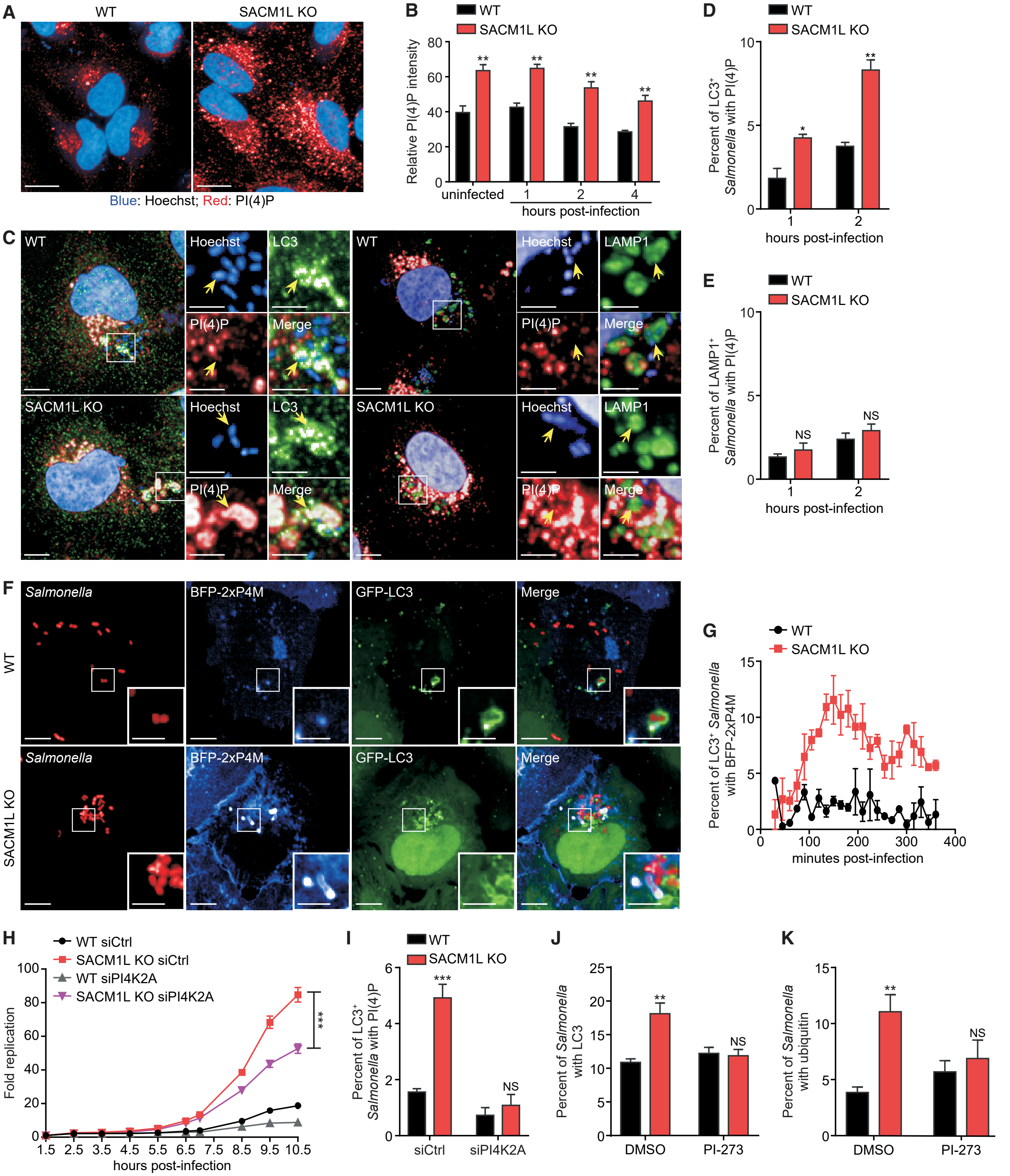

Figure 5. SAC1 regulates PI(4)P levels on *Salmonella*-containing autophagosomes

(A) Immunostaining of endogenous PI(4)P in WT and *SACM1L* KO cells. Hoechst dye shows nuclei. Scale bars represent 20 μm.

(B) Relative PI(4)P staining intensity in uninfected or *Salmonella*-infected WT and *SACM1L* KO cells at indicated times post-infection.

(C) Representative confocal images of PI(4)P staining on endogenous LC3^{+} or LAMP1^{+} *Salmonella* in WT and *SACM1L* KO cells at 2 h post-infection. Insets are boxed regions magnified (2.8×). Hoechst dye shows nuclei and *Salmonella*. Scale bars represent 10 μm in full images and 5 μm in insets.

(legend continued on next page)

(Figure 1A; Table S1). Similarly, we found that only knockdown of *PI4K2α* ameliorated the defect in bacterial restriction in *SACM1L* KO cells (Figures 5H and S5B–S5D). The percentage of *Salmonella*-containing autophagosomes with PI(4)P was also reduced in both WT and *SACM1L* KO cells after *PI4K2α* knockdown (Figure 5I). Furthermore, treatment with the PI4K2α-specific inhibitor PI-273 (Li et al., 2017) suppressed accumulation of LC3 and ubiquitin, which serve as markers for autophagosomal maturation, on *Salmonella* in *SACM1L* KO cells at 2 h post-infection (Figures 5J and 5K). These results indicate that regulation of PI(4)P levels on *Salmonella*-containing autophagosomes by PI4K2α and SAC1 is critical for efficient xenophagy.

SteA, a *Salmonella* effector protein, prevents maturation of *Salmonella*-containing autophagosomes in a PI(4)P-dependent manner

Given that our data support a specific role for SAC1 in xenophagy, we reasoned that *Salmonella* PI(4)P-binding proteins may contribute to the replicative advantages observed in *SACM1L* KO cells. SteA is a *Salmonella* type III secreted effector protein that specifically binds to PI(4)P, but its role in xenophagy is unclear (Domingues et al., 2016). By using a CFU assay, we found that replication of Δ*steA Salmonella*, unlike WT *Salmonella*, was restricted in SAC1-deficient cells (Figure 6A). Similar to WT *Salmonella*, however, Δ*steA Salmonella* replication increased in cells lacking NDP52 (Figure 6A). Loss of neither SAC1 nor SteA had detectable effects on *Salmonella* uptake as determined by CFU assay 1 h post-infection (Figure S6A). Importantly, reconstituting the Δ*steA* mutant with WT SteA, driven by its endogenous promoter or the stronger rpsM promoter, restored the *Salmonella* replicative advantage in *SACM1L* KO cells as compared to WT cells, whereas replication in *NDP52* KO cells remained unchanged (Figures 6B and 6C).

We next examined the subcellular localization of *Salmonella*-secreted SteA by infecting WT and *SACM1L* KO cells with a Δ*steA* mutant expressing V5-tagged SteA. SteA-V5 co-localized with both LC3$^+$ and LAMP1$^+$ *Salmonella*-containing compartments, indicating that SteA interacts with autophagosomes and SCVs (Figure 6D) as previously reported (Domingues et al., 2014). SAC1 loss increased the percentage of SteA-V5$^+$LC3$^+$ *Salmonella,* providing evidence that SteA localization correlates with PI(4)P levels on autophagosomal membranes (Figure 6E). Meanwhile, the percentage of SteA-V5$^+$LAMP1$^+$ *Salmonella* was comparable in WT and *SACM1L* KO cells (Figure 6F), consistent with PI(4)P levels on LAMP1$^+$ compartments (Figure 5E). Expression of SteA K36A-V5, which abolishes SteA binding to PI(4)P, dramatically decreased the recruitment of SteA to LC3$^+$ *Salmonella* (Figures S6B and S6C). Reconstituting the Δ*steA* mutant with WT SteA, but not SteA K36A or a GFP control, restored the replicative advantage of *Salmonella* in both *SACM1L* KO and WT cells, revealing the importance of SteA secretion to bacterial survival (Figure 6G). Unlike *SACM1L* KO cells, however, WT cells were able to suppress the replication of Δ*steA Salmonella*, supporting the importance of host PI(4)P regulation in xenophagy (Figure 6G). Moreover, only Δ*steA Salmonella* reconstituted with WT SteA induced LC3, ubiquitin, NDP52, and SQSTM1 accumulation in *SACM1L* KO cells and delayed maturation of autophagosomes (Figures 6H–6K and S6D), further indicating that SteA interferes with xenophagy in the presence of elevated PI(4)P levels.

To investigate how SteA impairs xenophagy, we transiently overexpressed SteA-V5 in mCherry-GFP-LC3 reporter cells. SteA-V5 co-localized with LC3 puncta and strongly impeded autophagosome acidification, as detected by increased levels of mCherry-LC3$^+$ and GFP-LC3$^+$ puncta in both WT and *SACM1L* KO cells compared with that of BFP-V5 control transfected cells (Figures 6L and 6M). These data indicate that SteA expression is sufficient to interfere with autophagosome-lysosomal fusion. Taken together, our findings demonstrate that SAC1 regulates the level of PI(4)P on *Salmonella*-containing autophagosomes, impairing the suppression of a host innate defense response by the *Salmonella*-secreted effector protein SteA.

DISCUSSION

Current models attribute specificity in selective autophagy to the binding of receptors to cargo, but a recent study revealed that NDP52 may also directly interact with lipid membranes during recruitment of autophagy initiation machinery (Shi et al., 2020). Moreover, the abundance of bacterial effectors that bind or modulate host phospholipids implicates their importance in host defense mechanisms (Haenssler and Isberg, 2011; Nachmias et al., 2019; Swart and Hilbi, 2020). Here, we investigated lipid membrane components as factors critical to xenophagy mechanisms.

From a targeted siRNA screen of lipid kinases and phosphatases, we identified the PI(4)P phosphatase SAC1 as a key regulator of intracellular bacterial replication. Both *SACM1L* knockdown and KO cells elevated bacterial replication. Re-expression of WT SAC1 restored bacterial replication restriction, whereas expression of the catalytically dead SAC1 C389S

(D and E) Percentage of LC3$^+$ (D) or LAMP1$^+$ (E) *Salmonella* also positive for PI(4)P at indicated times post-infection. For quantification, over 2,000 bacteria were analyzed.

(F and G) Representative confocal images (F) and quantification (G) of co-localization of BFP-2xP4M and GFP-LC3$^+$ *Salmonella* in WT and *SACM1L* KO cells. Insets are boxed regions magnified (2.5×). Scale bars represent 10 μm in full images and 5 μm in insets. Data were collected every 15 min for 6 h. For quantification, over 1,000 bacteria were analyzed.

(H) Fold change of luciferase-expressing *Salmonella* replication in WT and *SACM1L* KO cells transfected with control or *PI4K2α* siRNA for 48 h prior to infection. Luciferase levels were measured over time. Bacterial replication was normalized to baseline infection.

(I) Percentage of LC3$^+$ *Salmonella* associated with PI(4)P in WT and *SACM1L* KO cells transfected with control or *PI4K2α* siRNA 48 h prior to infection.

(J and K) Percentage of *Salmonella* associated with LC3 (J) and ubiquitin (K) in WT and *SACM1L* KO cells pretreated with DMSO or PI4K2α-specific inhibitor PI-273 (500 nM) for 1 h before infection, then fixed, and stained 2 h after infection. Unless indicated otherwise, over 500 cells were analyzed for quantification. Three independent experiments were analyzed using ANOVA (mean ± SEM). *p < 0.05, **p < 0.01, ***p < 0.001; NS, not significant.

See also Figure S5 and Table S2.

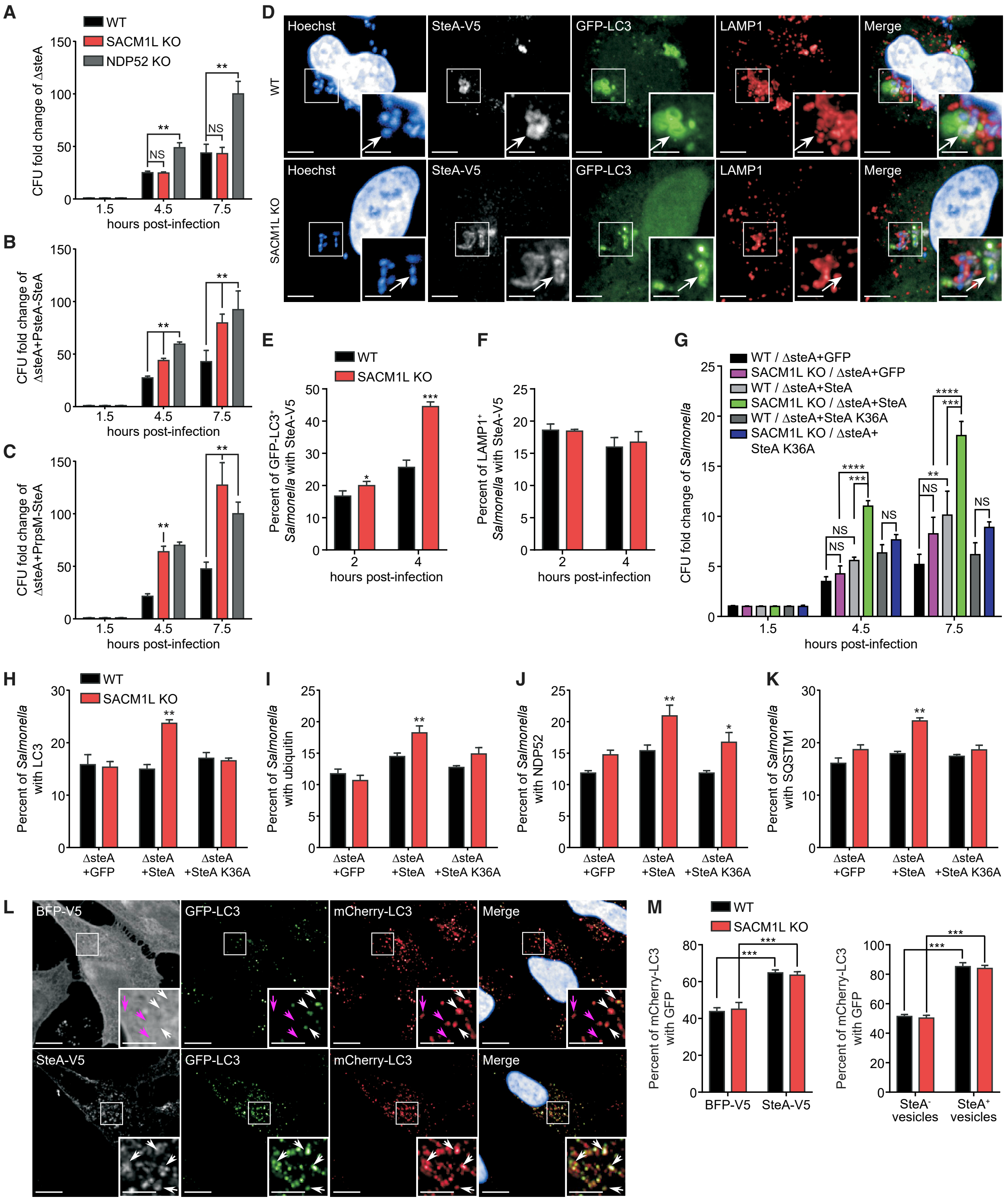

Figure 6. SteA, a *Salmonella* effector protein, prevents maturation of *Salmonella*-containing autophagosomes in a PI(4)P-dependent manner
(A–C) CFU fold change for the Δ*steA* mutant (A) and Δ*steA* mutant reconstituted with PsteA-SteA (B) or PrpsM-SteA (C) at indicated times post-infection of WT and *SACM1L* KO cells normalized to the 1.5-h time point.

(legend continued on next page)

mutant did not, indicating that PI(4)P phosphatase activity is required for host defense. Our study found that SAC1 loss had no detectable effect on non-selective autophagy, Parkin-dependent mitophagy, or aggrephagy, revealing a specific influence of PI(4)P regulation on xenophagy. Two recent siRNA studies reported contradictory roles for SAC1 in non-selective autophagosome maturation; however, in these studies, SAC1 protein levels and genetic rescue experiments were not performed to evaluate siRNA efficiency and off-target effects (Miao et al., 2020; Zhang et al., 2020). Our stable KO cells may also have compensated for the loss of SAC1 by using other phosphatases with less efficient activity on PI(4)P for constitutive autophagy functions.

As we did not detect defects in recognition or targeting of intracellular bacteria, we concluded that interactions between cargo receptors and membrane lipids were not responsible for the observed xenophagy defect. However, we found that SAC1 loss delayed accumulation of lysosomal markers on *Salmonella*-containing autophagosomes, indicating the importance of PI(4)P levels to autolysosomal maturation. Localization and activity of phosphoinositide lipids, regulators, and binding proteins collectively function to control subcellular membrane trafficking and interactions (Burke, 2018; Jeschke and Haas, 2018; Nishimura and Tooze, 2020). SAC1 is localized predominantly to the endoplasmic reticulum (ER) (Liu et al., 2009; Zewe et al., 2018). A recent study demonstrated that TMEM39A/SUSR2 regulates SAC1 trafficking between the ER and Golgi and the loss of SAC1 increases cellular PI(4)P (Miao et al., 2020), which is predominantly found on the plasma membrane, Golgi, and endosomal compartments (D'Angelo et al., 2008; Zewe et al., 2020).

We similarly found that cells lacking SAC1 expression had increased levels of cellular PI(4)P that co-localized with *Salmonella*-containing autophagosomes and had no detectable effect on PI(4)P associated with SCVs. This finding suggests that, in contrast to SCVs, *Salmonella*-containing autophagosomes sequester or stabilize PI(4)P (Domingues et al., 2016; Santos et al., 2015). Furthermore, reducing PI(4)P levels in *SACM1L* KO cells by either a *PI4K2α* siRNA or pharmacological inhibitor promoted maturation of *Salmonella*-containing autophagosomes and restricted bacterial replication. These results substantiate a role for SAC1-dependent regulation of PI(4)P in host defense.

Previous reports implicated PI4K2α regulation of PI(4)P in non-selective autophagosome maturation (Albanesi et al., 2015; Baba et al., 2019; Chen et al., 2018; Wang et al., 2015), whereas our results indicate that SAC1 phosphatase activity is necessary to modulate PI(4)P levels on *Salmonella*-containing autophagosomes for efficient autolysosomal fusion. Collectively, these findings indicate that both increased and decreased PI(4)P levels on autophagosomal membranes impair fusion with lysosomes.

Considering that our results suggested a xenophagy-specific role for SAC1, we investigated the contribution of *Salmonella* to delaying lysosomal fusion as a result of PI(4)P accumulation on *Salmonella*-containing autophagosomes. We evaluated SteA, a *Salmonella*-secreted effector that binds specifically to PI(4)P and localizes to PI(4)P-rich SCVs when ectopically expressed in infected cells (Domingues et al., 2016; Matsuda et al., 2019). Importantly, both WT and *SACM1L* KO cells were able to restrict replication of *Salmonella* lacking SteA to different extents, supporting a role for SteA in replication. Meanwhile, *SACM1L* KO cells infected with *Salmonella* expressing SteA displayed increased bacterial replication and delayed degradation of autophagy markers on bacteria. We propose that the loss of SAC1 increases PI(4)P levels on *Salmonella*-containing autophagosomes, thereby promoting SteA accumulation, which impairs lysosomal fusion and results in increased bacterial replication. Based on our data, higher levels of SteA expression driven by a strong bacterial promoter increased bacterial numbers compared with endogenous SteA expression, further supporting that SteA accumulation on autophagosomal membranes facilitates *Salmonella* replication. Moreover, ectopic expression of SteA in either WT or *SACM1L* KO cells localized to autophagosomes and blocked basal autophagic flux, confirming that SteA plays a direct role in preventing lysosomal fusion.

Using live cell imaging, we observed an increase in *Salmonella* bacteria replicating in the cytosol of cells lacking SAC1 despite being targeted by autophagy machinery shortly after infection. These bacteria may account for the robust replication phenotype as compared to the modest autophagolysosome maturation phenotype. Recently, the lipid transporter and PI(4)P-binding protein OSBP1 was found to interact with SCVs through *Salmonella* effectors SseJ and SseL. Together, these proteins stabilize the SCV, and the loss of either the effectors or OSBP1 increases cytosolic bacteria (Kolodziejek et al., 2019). Dysregulation of PI(4)P may alter SCV stability in cells lacking SAC1, which may also contribute to the increase in bacterial replication; however, we did not observe a significant change in SCV integrity upon SAC1 loss.

Intracellular bacteria are known to manipulate host cell processes to develop replication-competent niches or avoid defense mechanisms (Asrat et al., 2014; Case and Samuel, 2016; Kimmey and Stallings, 2016; Pao and Rape, 2019). *Salmonella*, as well as other pathogens, produces an array of effector proteins—with functions ranging from inducing uptake to

(D) Representative confocal images of *Salmonella* associated with SteA-V5 (white arrows) in WT and *SACM1L* KO cells at 2 h post-infection with Δ*steA* reconstituted with SteA-V5. Insets are boxed regions magnified (2×). Scale bars represent 10 μm in full images and 5 μm in insets.

(E and F) Percentage of LC3$^+$ (E) or LAMP1$^+$ (F) *Salmonella* associated with SteA-V5 at indicated times post-infection.

(G) CFU fold changes normalized to the 1.5-h time points for *Salmonella* strains at indicated times post-infection of WT and *SACM1L* KO cells.

(H–K) Percentage of LC3$^+$ (H), ubiquitin$^+$ (I), NDP52$^+$ (J), or SQSTM1$^+$ (K) *Salmonella* in WT and *SACM1L* KO cells at 2 h post-infection.

(L and M) Representative confocal images (L) and quantification (M) of mCherry-GFP-LC3 in WT and *SACM1L* KO cells transiently expressing BFP-V5 or SteA-V5 after 24 h. Hoechst shows nuclei. Scale bars represent 5 μm. Arrows indicate mCherry$^+$GFP$^+$ (white) and mCherry$^+$GFP$^-$ (magenta) vesicles. For all quantifications, over 500 cells were analyzed. Three independent experiments were analyzed using ANOVA (mean ± SEM). *$p < 0.05$, **$p < 0.01$, ***$p < 0.001$, ****$p < 0.0001$; NS, not significant.

See also Figure S6.

developing a replication-competent vacuole (Cardenal-Muñoz and Ramos-Morales, 2011)—that act both redundantly and cooperatively to ensure survival (Azimi et al., 2020; Ghosh and O'Connor, 2017). Our results highlight the importance of innate host defense to restrict bacterial replication and the role of bacterial factors in intracellular survival. In WT cells, SteA had a modest effect on bacterial survival; however, PI(4)P dysregulation shifted the advantage to *Salmonella.* Recent reports described the role of SopF, another phosphoinositide-binding effector, in promoting SCV membrane integrity as well as potently blocking xenophagy by modifying ATP6V0C, a vacuolar ATPase on the SCV, to prevent recruitment of the core autophagy protein ATG16L1 (Fischer et al., 2020; Lau et al., 2019; Xu et al., 2019). Our data not only establish the importance of SAC1-dependent PI(4)P regulation in xenophagy-specific autophagolysosome maturation but also expand our understanding of how *Salmonella* SteA PI(4)P binding supports intracellular replication by interfering with a host defense mechanism.

STAR★METHODS

Detailed methods are provided in the online version of this paper and include the following:

- KEY RESOURCES TABLE
- RESOURCE AVAILABILITY
  - Lead contact
  - Materials availability
  - Data and code availability
- EXPERIMENTAL MODEL AND SUBJECT DETAILS
  - Cell line culture and small interfering RNA (siRNA) knockdown
  - Vector construction
  - Bacterial strains
- METHOD DETAILS
  - Generation of CRISPR knockout cells
  - Bacterial infection assays
  - Chloroquine (CHQ) resistance assay
  - X-light-mCherry bacterial assay
  - Immunofluorescence
  - Microscopy
  - Quantitative PCR
- QUANTIFICATION AND STATISTICAL ANALYSIS

SUPPLEMENTAL INFORMATION

Supplemental information can be found online at https://doi.org/10.1016/j.celrep.2021.109434.

ACKNOWLEDGMENTS

We thank Drs. Luís Jaime Mota at NOVA University of Lisbon for the generous gift of *S*. Typhimurium Δ*steA* deletion, Tamotsu Yoshimori at Osaka University for the pEGFP-hGal3 plasmid, Serge Mostowy at the London School of Hygiene and Tropical Medicine for the pKB269 x-light mCherry plasmid, Leigh Baxt for generation of x-light mCherry-expressing *Salmonella*, Herbert (Skip) Virgin and the late Beth Levine for invaluable feedback, Theresa Reimels for help with manuscript preparation, and Jacques Deguine for comments. This work was funded by the National Institutes of Health (U19AI109725, U19AI142784, R01DK117263, and R01DK097485 to R.J.X.).

AUTHOR CONTRIBUTIONS

Conceptualization, K.L., K.L.C., and R.J.X.; investigation, K.L.; formal analysis, K.L. and L.K.; writing, original draft, K.L. and K.L.C.; writing, reviewing & editing, K.L., D.B.G., K.L.C., and R.J.X.; visualization, K.L. and L.K.; funding acquisition, R.J.X.

DECLARATION OF INTERESTS

R.J.X. is co-founder of Jnana Therapeutics and Celsius Therapeutics. These organizations had no roles in this study.

Received: October 21, 2020
Revised: December 21, 2020
Accepted: July 1, 2021
Published: July 27, 2021

REFERENCES

Albanesi, J., Wang, H., Sun, H.Q., Levine, B., and Yin, H. (2015). GABARAP-mediated targeting of PI4K2A/PI4KIIα to autophagosomes regulates PtdIns4P-dependent autophagosome-lysosome fusion. Autophagy *11*, 2127–2129.

Asrat, S., de Jesús, D.A., Hempstead, A.D., Ramabhadran, V., and Isberg, R.R. (2014). Bacterial pathogen manipulation of host membrane trafficking. Annu. Rev. Cell Dev. Biol. *30*, 79–109.

Azimi, T., Zamirnasta, M., Sani, M.A., Soltan Dallal, M.M., and Nasser, A. (2020). Molecular Mechanisms of *Salmonella* Effector Proteins: A Comprehensive Review. Infect. Drug Resist. *13*, 11–26.

Baba, T., Toth, D.J., Sengupta, N., Kim, Y.J., and Balla, T. (2019). Phosphatidylinositol 4,5-bisphosphate controls Rab7 and PLEKHM1 membrane cycling during autophagosome-lysosome fusion. EMBO J. *38*, e100312.

Barnett, T.C., Liebl, D., Seymour, L.M., Gillen, C.M., Lim, J.Y., Larock, C.N., Davies, M.R., Schulz, B.L., Nizet, V., Teasdale, R.D., and Walker, M.J. (2013). The globally disseminated M1T1 clone of group A Streptococcus evades autophagy for intracellular replication. Cell Host Microbe *14*, 675–682.

Benjamin, J.L., Sumpter, R., Jr., Levine, B., and Hooper, L.V. (2013). Intestinal epithelial autophagy is essential for host defense against invasive bacteria. Cell Host Microbe *13*, 723–734.

Bento, C.F., Renna, M., Ghislat, G., Puri, C., Ashkenazi, A., Vicinanza, M., Menzies, F.M., and Rubinsztein, D.C. (2016). Mammalian Autophagy: How Does It Work? Annu. Rev. Biochem. *85*, 685–713.

Birmingham, C.L., Smith, A.C., Bakowski, M.A., Yoshimori, T., and Brumell, J.H. (2006). Autophagy controls Salmonella infection in response to damage to the Salmonella-containing vacuole. J. Biol. Chem. *281*, 11374–11383.

Bjørkøy, G., Lamark, T., Brech, A., Outzen, H., Perander, M., Overvatn, A., Stenmark, H., and Johansen, T. (2005). p62/SQSTM1 forms protein aggregates degraded by autophagy and has a protective effect on huntingtin-induced cell death. J. Cell Biol. *171*, 603–614.

Burke, J.E. (2018). Structural Basis for Regulation of Phosphoinositide Kinases and Their Involvement in Human Disease. Mol. Cell *71*, 653–673.

Cardenal-Muñoz, E., and Ramos-Morales, F. (2011). Analysis of the expression, secretion and translocation of the Salmonella enterica type III secretion system effector SteA. PLoS One *6*, e26930.

Case, E.D.R., and Samuel, J.E. (2016). Contrasting Lifestyles Within the Host Cell. Microbiol. Spectr. *4*, 0014–2015. https://doi.org/10.1128/microbiolspec.VMBF.

Castanheira, S., and García-Del Portillo, F. (2017). *Salmonella* Populations inside Host Cells. Front. Cell. Infect. Microbiol. *7*, 432.

Chen, J., and Chen, Z.J. (2018). PtdIns4P on dispersed trans-Golgi network mediates NLRP3 inflammasome activation. Nature *564*, 71–76.

Chen, C.S., Chen, W.N., Zhou, M., Arttamangkul, S., and Haugland, R.P. (2000). Probing the cathepsin D using a BODIPY FL-pepstatin A: applications

in fluorescence polarization and microscopy. J. Biochem. Biophys. Methods *42*, 137–151.

Chen, Y., Sun, H.Q., Eichorst, J.P., Albanesi, J.P., Yin, H., and Mueller, J.D. (2018). Comobility of GABARAP and Phosphatidylinositol 4-Kinase 2A on Cytoplasmic Vesicles. Biochemistry *57*, 3556–3559.

Choy, A., Dancourt, J., Mugo, B., O'Connor, T.J., Isberg, R.R., Melia, T.J., and Roy, C.R. (2012). The Legionella effector RavZ inhibits host autophagy through irreversible Atg8 deconjugation. Science *338*, 1072–1076.

Clarke, A.J., and Simon, A.K. (2019). Autophagy in the renewal, differentiation and homeostasis of immune cells. Nat. Rev. Immunol. *19*, 170–183.

Clayton, E.L., Minogue, S., and Waugh, M.G. (2013). Mammalian phosphatidylinositol 4-kinases as modulators of membrane trafficking and lipid signaling networks. Prog. Lipid Res. *52*, 294–304.

Conway, K.L., Kuballa, P., Song, J.H., Patel, K.K., Castoreno, A.B., Yilmaz, O.H., Jijon, H.B., Zhang, M., Aldrich, L.N., Villablanca, E.J., et al. (2013). Atg16l1 is required for autophagy in intestinal epithelial cells and protection of mice from Salmonella infection. Gastroenterology *145*, 1347–1357.

D'Angelo, G., Vicinanza, M., Di Campli, A., and De Matteis, M.A. (2008). The multiple roles of PtdIns(4)P—not just the precursor of PtdIns(4,5)P2. J. Cell Sci. *121*, 1955–1963.

Dall'Armi, C., Devereaux, K.A., and Di Paolo, G. (2013). The role of lipids in the control of autophagy. Curr. Biol. *23*, R33–R45.

de la Ballina, L.R., Munson, M.J., and Simonsen, A. (2020). Lipids and Lipid-Binding Proteins in Selective Autophagy. J. Mol. Biol. *432*, 135–159.

De Tito, S., Hervás, J.H., van Vliet, A.R., and Tooze, S.A. (2020). The Golgi as an Assembly Line to the Autophagosome. Trends Biochem. Sci. *45*, 484–496.

Del Bel, L.M., and Brill, J.A. (2018). Sac1, a lipid phosphatase at the interface of vesicular and nonvesicular transport. Traffic *19*, 301–318.

Deosaran, E., Larsen, K.B., Hua, R., Sargent, G., Wang, Y., Kim, S., Lamark, T., Jauregui, M., Law, K., Lippincott-Schwartz, J., et al. (2013). NBR1 acts as an autophagy receptor for peroxisomes. J. Cell Sci. *126*, 939–952.

Dickson, E.J., and Hille, B. (2019). Understanding phosphoinositides: rare, dynamic, and essential membrane phospholipids. Biochem. J. *476*, 1–23.

Domingues, L., Holden, D.W., and Mota, L.J. (2014). The Salmonella effector SteA contributes to the control of membrane dynamics of Salmonella-containing vacuoles. Infect. Immun. *82*, 2923–2934.

Domingues, L., Ismail, A., Charro, N., Rodríguez-Escudero, I., Holden, D.W., Molina, M., Cid, V.J., and Mota, L.J. (2016). The Salmonella effector SteA binds phosphatidylinositol 4-phosphate for subcellular targeting within host cells. Cell. Microbiol. *18*, 949–969.

Dooley, H.C., Razi, M., Polson, H.E., Girardin, S.E., Wilson, M.I., and Tooze, S.A. (2014). WIPI2 links LC3 conjugation with PI3P, autophagosome formation, and pathogen clearance by recruiting Atg12-5-16L1. Mol. Cell *55*, 238–252.

Figueira, R., Watson, K.G., Holden, D.W., and Helaine, S. (2013). Identification of salmonella pathogenicity island-2 type III secretion system effectors involved in intramacrophage replication of S. enterica serovar typhimurium: implications for rational vaccine design. mBio *4*, e00065.

Fischer, T.D., Wang, C., Padman, B.S., Lazarou, M., and Youle, R.J. (2020). STING induces LC3B lipidation onto single-membrane vesicles via the V-ATPase and ATG16L1-WD40 domain. J. Cell Biol. *219*, e202009128.

Fracchiolla, D., Chang, C., Hurley, J.H., and Martens, S. (2020). A PI3K-WIPI2 positive feedback loop allosterically activates LC3 lipidation in autophagy. J. Cell Biol. *219*, e201912098.

Gatica, D., Lahiri, V., and Klionsky, D.J. (2018). Cargo recognition and degradation by selective autophagy. Nat. Cell Biol. *20*, 233–242.

Ghosh, S., and O'Connor, T.J. (2017). Beyond Paralogs: The Multiple Layers of Redundancy in Bacterial Pathogenesis. Front. Cell. Infect. Microbiol. *7*, 467.

Gomez-Valero, L., Rusniok, C., Carson, D., Mondino, S., Pérez-Cobas, A.E., Rolando, M., Pasricha, S., Reuter, S., Demirtas, J., Crumbach, J., et al. (2019). More than 18,000 effectors in the *Legionella* genus genome provide multiple, independent combinations for replication in human cells. Proc. Natl. Acad. Sci. USA *116*, 2265–2273.

Haenssler, E., and Isberg, R.R. (2011). Control of host cell phosphorylation by legionella pneumophila. Front. Microbiol. *2*, 64.

Hammond, G.R., Schiavo, G., and Irvine, R.F. (2009). Immunocytochemical techniques reveal multiple, distinct cellular pools of PtdIns4P and PtdIns(4,5)P(2). Biochem. J. *422*, 23–35.

Hansen, T.E., and Johansen, T. (2011). Following autophagy step by step. BMC Biol. *9*, 39.

Hashimoto, Y., Shirane, M., and Nakayama, K.I. (2018). TMEM55B contributes to lysosomal homeostasis and amino acid-induced mTORC1 activation. Genes Cells *23*, 418–434.

Heath, R.J., Goel, G., Baxt, L.A., Rush, J.S., Mohanan, V., Paulus, G.L.C., Jani, V., Lassen, K.G., and Xavier, R.J. (2016). RNF166 Determines Recruitment of Adaptor Proteins during Antibacterial Autophagy. Cell Rep. *17*, 2183–2194.

Heo, J.M., Ordureau, A., Paulo, J.A., Rinehart, J., and Harper, J.W. (2015). The PINK1-PARKIN Mitochondrial Ubiquitylation Pathway Drives a Program of OPTN/NDP52 Recruitment and TBK1 Activation to Promote Mitophagy. Mol. Cell *60*, 7–20.

Hoiseth, S.K., and Stocker, B.A. (1981). Aromatic-dependent Salmonella typhimurium are non-virulent and effective as live vaccines. Nature *291*, 238–239.

Horenkamp, F.A., Kauffman, K.J., Kohler, L.J., Sherwood, R.K., Krueger, K.P., Shteyn, V., Roy, C.R., Melia, T.J., and Reinisch, K.M. (2015). The Legionella Anti-autophagy Effector RavZ Targets the Autophagosome via PI3P- and Curvature-Sensing Motifs. Dev. Cell *34*, 569–576.

Huang, J., and Brumell, J.H. (2014). Bacteria-autophagy interplay: a battle for survival. Nat. Rev. Microbiol. *12*, 101–114.

Hubber, A., Arasaki, K., Nakatsu, F., Hardiman, C., Lambright, D., De Camilli, P., Nagai, H., and Roy, C.R. (2014). The machinery at endoplasmic reticulum-plasma membrane contact sites contributes to spatial regulation of multiple Legionella effector proteins. PLoS Pathog. *10*, e1004222.

Huett, A., Heath, R.J., Begun, J., Sassi, S.O., Baxt, L.A., Vyas, J.M., Goldberg, M.B., and Xavier, R.J. (2012). The LRR and RING domain protein LRSAM1 is an E3 ligase crucial for ubiquitin-dependent autophagy of intracellular Salmonella Typhimurium. Cell Host Microbe *12*, 778–790.

Jeschke, A., and Haas, A. (2018). Sequential actions of phosphatidylinositol phosphates regulate phagosome-lysosome fusion. Mol. Biol. Cell *29*, 452–465.

Kehl, A., Göser, V., Reuter, T., Liss, V., Franke, M., John, C., Richter, C.P., Deiwick, J., and Hensel, M. (2020). A trafficome-wide RNAi screen reveals deployment of early and late secretory host proteins and the entire late endo-/lysosomal vesicle fusion machinery by intracellular Salmonella. PLoS Pathog. *16*, e1008220.

Kim, B.W., Kwon, D.H., and Song, H.K. (2016). Structure biology of selective autophagy receptors. BMB Rep. *49*, 73–80.

Kimmey, J.M., and Stallings, C.L. (2016). Bacterial Pathogens versus Autophagy: Implications for Therapeutic Interventions. Trends Mol. Med. *22*, 1060–1076.

Kirkin, V., and Rogov, V.V. (2019). A Diversity of Selective Autophagy Receptors Determines the Specificity of the Autophagy Pathway. Mol. Cell *76*, 268–285.

Knodler, L.A., Nair, V., and Steele-Mortimer, O. (2014). Quantitative assessment of cytosolic Salmonella in epithelial cells. PLoS One *9*, e84681.

Kolodziejek, A.M., Altura, M.A., Fan, J., Petersen, E.M., Cook, M., Brzovic, P.S., and Miller, S.I. (2019). Salmonella Translocated Effectors Recruit OSBP1 to the Phagosome to Promote Vacuolar Membrane Integrity. Cell Rep. *27*, 2147–2156.e5.

Krokowski, S., Lobato-Márquez, D., and Mostowy, S. (2018). Mitochondria promote septin assembly into cages that entrap Shigella for autophagy. Autophagy *14*, 913–914.

Kubori, T., Bui, X.T., Hubber, A., and Nagai, H. (2017). *Legionella* RavZ Plays a Role in Preventing Ubiquitin Recruitment to Bacteria-Containing Vacuoles. Front. Cell. Infect. Microbiol. *7*, 384.

Lane, K., Andres-Terre, M., Kudo, T., Monack, D.M., and Covert, M.W. (2019). Escalating Threat Levels of Bacterial Infection Can Be Discriminated by Distinct MAPK and NF-κB Signaling Dynamics in Single Host Cells. Cell Syst. *8*, 183–196.e4.

Lau, N., Haeberle, A.L., O'Keeffe, B.J., Latomanski, E.A., Celli, J., Newton, H.J., and Knodler, L.A. (2019). SopF, a phosphoinositide binding effector, promotes the stability of the nascent Salmonella-containing vacuole. PLoS Pathog. *15*, e1007959.

Lazarou, M., Sliter, D.A., Kane, L.A., Sarraf, S.A., Wang, C., Burman, J.L., Sideris, D.P., Fogel, A.I., and Youle, R.J. (2015). The ubiquitin kinase PINK1 recruits autophagy receptors to induce mitophagy. Nature *524*, 309–314.

Levin-Konigsberg, R., Montaño-Rendón, F., Keren-Kaplan, T., Li, R., Ego, B., Mylvaganam, S., DiCiccio, J.E., Trimble, W.S., Bassik, M.C., Bonifacino, J.S., et al. (2019). Phagolysosome resolution requires contacts with the endoplasmic reticulum and phosphatidylinositol-4-phosphate signalling. Nat. Cell Biol. *21*, 1234–1247.

Levine, B., and Kroemer, G. (2008). Autophagy in the pathogenesis of disease. Cell *132*, 27–42.

Li, J., Gao, Z., Zhao, D., Zhang, L., Qiao, X., Zhao, Y., Ding, H., Zhang, P., Lu, J., Liu, J., et al. (2017). PI-273, a Substrate-Competitive, Specific Small-Molecule Inhibitor of PI4KIIα, Inhibits the Growth of Breast Cancer Cells. Cancer Res. *77*, 6253–6266.

Liu, Y., Boukhelifa, M., Tribble, E., Morin-Kensicki, E., Uetrecht, A., Bear, J.E., and Bankaitis, V.A. (2008). The Sac1 phosphoinositide phosphatase regulates Golgi membrane morphology and mitotic spindle organization in mammals. Mol. Biol. Cell *19*, 3080–3096.

Liu, Y., Boukhelifa, M., Tribble, E., and Bankaitis, V.A. (2009). Functional studies of the mammalian Sac1 phosphoinositide phosphatase. Adv. Enzyme Regul. *49*, 75–86.

Liu, L., Feng, D., Chen, G., Chen, M., Zheng, Q., Song, P., Ma, Q., Zhu, C., Wang, R., Qi, W., et al. (2012). Mitochondrial outer-membrane protein FUNDC1 mediates hypoxia-induced mitophagy in mammalian cells. Nat. Cell Biol. *14*, 177–185.

Luo, X., Wasilko, D.J., Liu, Y., Sun, J., Wu, X., Luo, Z.Q., and Mao, Y. (2015). Structure of the Legionella Virulence Factor, SidC Reveals a Unique PI(4)P-Specific Binding Domain Essential for Its Targeting to the Bacterial Phagosome. PLoS Pathog. *11*, e1004965.

Maejima, I., Takahashi, A., Omori, H., Kimura, T., Takabatake, Y., Saitoh, T., Yamamoto, A., Hamasaki, M., Noda, T., Isaka, Y., and Yoshimori, T. (2013). Autophagy sequesters damaged lysosomes to control lysosomal biogenesis and kidney injury. EMBO J. *32*, 2336–2347.

Mancias, J.D., Wang, X., Gygi, S.P., Harper, J.W., and Kimmelman, A.C. (2014). Quantitative proteomics identifies NCOA4 as the cargo receptor mediating ferritinophagy. Nature *509*, 105–109.

Matsuda, S., Haneda, T., Saito, H., Miki, T., and Okada, N. (2019). *Salmonella enterica* Effectors SifA, SpvB, SseF, SseJ, and SteA Contribute to Type III Secretion System 1-Independent Inflammation in a Streptomycin-Pretreated Mouse Model of Colitis. Infect. Immun. *87*, e00872-18.

Mesquita, F.S., Thomas, M., Sachse, M., Santos, A.J., Figueira, R., and Holden, D.W. (2012). The Salmonella deubiquitinase SseL inhibits selective autophagy of cytosolic aggregates. PLoS Pathog. *8*, e1002743.

Miao, G., Zhang, Y., Chen, D., and Zhang, H. (2020). The ER-Localized Transmembrane Protein TMEM39A/SUSR2 Regulates Autophagy by Controlling the Trafficking of the PtdIns(4)P Phosphatase SAC1. Mol. Cell *77*, 618–632.e5.

Mitchell, G., Cheng, M.I., Chen, C., Nguyen, B.N., Whiteley, A.T., Kianian, S., Cox, J.S., Green, D.R., McDonald, K.L., and Portnoy, D.A. (2018). *Listeria monocytogenes* triggers noncanonical autophagy upon phagocytosis, but avoids subsequent growth-restricting xenophagy. Proc. Natl. Acad. Sci. USA *115*, E210–E217.

Mizushima, N., and Komatsu, M. (2011). Autophagy: renovation of cells and tissues. Cell *147*, 728–741.

Morioka, S., Nigorikawa, K., Okada, E., Tanaka, Y., Kasuu, Y., Yamada, M., Kofuji, S., Takasuga, S., Nakanishi, H., Sasaki, T., and Hazeki, K. (2018). TMEM55a localizes to macrophage phagosomes to downregulate phagocytosis. J. Cell Sci. *131*, jcs213272.

Nachmias, N., Zusman, T., and Segal, G. (2019). Study of *Legionella* Effector Domains Revealed Novel and Prevalent Phosphatidylinositol 3-Phosphate Binding Domains. Infect. Immun. *87*, e00153-19.

Nakada-Tsukui, K., Watanabe, N., Maehama, T., and Nozaki, T. (2019). Phosphatidylinositol Kinases and Phosphatases in *Entamoeba histolytica*. Front. Cell. Infect. Microbiol. *9*, 150.

Nakatogawa, H. (2020). Mechanisms governing autophagosome biogenesis. Nat. Rev. Mol. Cell Biol. *21*, 439–458.

Nishimura, T., and Tooze, S.A. (2020). Emerging roles of ATG proteins and membrane lipids in autophagosome formation. Cell Discov. *6*, 32.

Pao, K.C., and Rape, M. (2019). Tug of War in the Xenophagy World. Trends Cell Biol. *29*, 767–769.

Paz, I., Sachse, M., Dupont, N., Mounier, J., Cederfur, C., Enninga, J., Leffler, H., Poirier, F., Prevost, M.C., Lafont, F., and Sansonetti, P. (2010). Galectin-3, a marker for vacuole lysis by invasive pathogens. Cell. Microbiol. *12*, 530–544.

Pham, H.Q., Yoshioka, K., Mohri, H., Nakata, H., Aki, S., Ishimaru, K., Takuwa, N., and Takuwa, Y. (2018). MTMR4, a phosphoinositide-specific 3′-phosphatase, regulates TFEB activity and the endocytic and autophagic pathways. Genes Cells *23*, 670–687.

Polajnar, M., Dietz, M.S., Heilemann, M., and Behrends, C. (2017). Expanding the host cell ubiquitylation machinery targeting cytosolic *Salmonella*. EMBO Rep. *18*, 1572–1585.

Polson, H.E., de Lartigue, J., Rigden, D.J., Reedijk, M., Urbé, S., Clague, M.J., and Tooze, S.A. (2010). Mammalian Atg18 (WIPI2) localizes to omegasome-anchored phagophores and positively regulates LC3 lipidation. Autophagy *6*, 506–522.

Ravenhill, B.J., Boyle, K.B., von Muhlinen, N., Ellison, C.J., Masson, G.R., Otten, E.G., Foeglein, A., Williams, R., and Randow, F. (2019). The Cargo Receptor NDP52 Initiates Selective Autophagy by Recruiting the ULK Complex to Cytosol-Invading Bacteria. Mol. Cell *74*, 320–329.e6.

Rioux, J.D., Xavier, R.J., Taylor, K.D., Silverberg, M.S., Goyette, P., Huett, A., Green, T., Kuballa, P., Barmada, M.M., Datta, L.W., et al. (2007). Genome-wide association study identifies new susceptibility loci for Crohn disease and implicates autophagy in disease pathogenesis. Nat. Genet. *39*, 596–604.

Sacco, F., Perfetto, L., Castagnoli, L., and Cesareni, G. (2012). The human phosphatase interactome: An intricate family portrait. FEBS Lett. *586*, 2732–2739.

Santiago-Tirado, F.H., and Bretscher, A. (2011). Membrane-trafficking sorting hubs: cooperation between PI4P and small GTPases at the trans-Golgi network. Trends Cell Biol. *21*, 515–525.

Santos, J.C., Duchateau, M., Fredlund, J., Weiner, A., Mallet, A., Schmitt, C., Matondo, M., Hourdel, V., Chamot-Rooke, J., and Enninga, J. (2015). The COPII complex and lysosomal VAMP7 determine intracellular Salmonella localization and growth. Cell. Microbiol. *17*, 1699–1720.

Sarraf, S.A., Shah, H.V., Kanfer, G., Pickrell, A.M., Holtzclaw, L.A., Ward, M.E., and Youle, R.J. (2020). Loss of TAX1BP1-Directed Autophagy Results in Protein Aggregate Accumulation in the Brain. Mol. Cell *80*, 779–795.e10.

Sasaki, T., Takasuga, S., Sasaki, J., Kofuji, S., Eguchi, S., Yamazaki, M., and Suzuki, A. (2009). Mammalian phosphoinositide kinases and phosphatases. Prog. Lipid Res. *48*, 307–343.

Schroeder, G.N. (2018). The Toolbox for Uncovering the Functions of *Legionella* Dot/Icm Type IVb Secretion System Effectors: Current State and Future Directions. Front. Cell. Infect. Microbiol. *7*, 528.

Shaid, S., Brandts, C.H., Serve, H., and Dikic, I. (2013). Ubiquitination and selective autophagy. Cell Death Differ. *20*, 21–30.

Sharma, V., Verma, S., Seranova, E., Sarkar, S., and Kumar, D. (2018). Selective Autophagy and Xenophagy in Infection and Disease. Front. Cell Dev. Biol. *6*, 147.

CellPress
OPEN ACCESS

Shi, X., Chang, C., Yokom, A.L., Jensen, L.E., and Hurley, J.H. (2020). The autophagy adaptor NDP52 and the FIP200 coiled-coil allosterically activate ULK1 complex membrane recruitment. eLife *9*, e59099.

Simonsen, A., and Tooze, S.A. (2009). Coordination of membrane events during autophagy by multiple class III PI3-kinase complexes. J. Cell Biol. *186*, 773–782.

Sirianni, A., Krokowski, S., Lobato-Márquez, D., Buranyi, S., Pfanzelter, J., Galea, D., Willis, A., Culley, S., Henriques, R., Larrouy-Maumus, G., et al. (2016). Mitochondria mediate septin cage assembly to promote autophagy of Shigella. EMBO Rep. *17*, 1029–1043.

Swart, A.L., and Hilbi, H. (2020). Phosphoinositides and the Fate of *Legionella* in Phagocytes. Front. Immunol. *11*, 25.

Takemasu, S., Nigorikawa, K., Yamada, M., Tsurumi, G., Kofuji, S., Takasuga, S., and Hazeki, K. (2019). Phosphorylation of TMEM55B by Erk/MAPK regulates lysosomal positioning. J. Biochem. *166*, 175–185.

Tattoli, I., Sorbara, M.T., Yang, C., Tooze, S.A., Philpott, D.J., and Girardin, S.E. (2013). Listeria phospholipases subvert host autophagic defenses by stalling pre-autophagosomal structures. EMBO J. *32*, 3066–3078.

Teo, W.X., Kerr, M.C., and Teasdale, R.D. (2016). MTMR4 Is Required for the Stability of the Salmonella-Containing Vacuole. Front. Cell. Infect. Microbiol. *6*, 91.

Thurston, T.L., Wandel, M.P., von Muhlinen, N., Foeglein, A., and Randow, F. (2012). Galectin 8 targets damaged vesicles for autophagy to defend cells against bacterial invasion. Nature *482*, 414–418.

Tuli, A., and Sharma, M. (2019). How to do business with lysosomes: Salmonella leads the way. Curr. Opin. Microbiol. *47*, 1–7.

Valdivia, R.H., and Falkow, S. (1996). Bacterial genetics by flow cytometry: rapid isolation of Salmonella typhimurium acid-inducible promoters by differential fluorescence induction. Mol. Microbiol. *22*, 367–378.

Vargas, J.N.S., Wang, C., Bunker, E., Hao, L., Maric, D., Schiavo, G., Randow, F., and Youle, R.J. (2019). Spatiotemporal Control of ULK1 Activation by NDP52 and TBK1 during Selective Autophagy. Mol. Cell *74*, 347–362.e6.

Venditti, R., Masone, M.C., Rega, L.R., Di Tullio, G., Santoro, M., Polishchuk, E., Serrano, I.C., Olkkonen, V.M., Harada, A., Medina, D.L., et al. (2019). The activity of Sac1 across ER-TGN contact sites requires the four-phosphate-adaptor-protein-1. J. Cell Biol. *218*, 783–797.

Wang, J., Chen, J., Enns, C.A., and Mayinger, P. (2013). The first transmembrane domain of lipid phosphatase SAC1 promotes Golgi localization. PLoS One *8*, e71112.

Wang, H., Sun, H.Q., Zhu, X., Zhang, L., Albanesi, J., Levine, B., and Yin, H. (2015). GABARAPs regulate PI4P-dependent autophagosome:lysosome fusion. Proc. Natl. Acad. Sci. USA *112*, 7015–7020.

Wasilko, D.J., and Mao, Y. (2016). Exploiting the ubiquitin and phosphoinositide pathways by the Legionella pneumophila effector, SidC. Curr. Genet. *62*, 105–108.

Weber, S., Steiner, B., Welin, A., and Hilbi, H. (2018). *Legionella*-Containing Vacuoles Capture PtdIns(4)*P*-Rich Vesicles Derived from the Golgi Apparatus. mBio *9*, e02420-18.

Willett, R., Martina, J.A., Zewe, J.P., Wills, R., Hammond, G.R.V., and Puertollano, R. (2017). TFEB regulates lysosomal positioning by modulating TMEM55B expression and JIP4 recruitment to lysosomes. Nat. Commun. *8*, 1580.

Xiao, Y., and Cai, W. (2020). Autophagy and Bacterial Infection. Adv. Exp. Med. Biol. *1207*, 413–423.

Xu, Y., Zhou, P., Cheng, S., Lu, Q., Nowak, K., Hopp, A.K., Li, L., Shi, X., Zhou, Z., Gao, W., et al. (2019). A Bacterial Effector Reveals the V-ATPase-ATG16L1 Axis that Initiates Xenophagy. Cell *178*, 552–566.e20.

Yamano, K., Fogel, A.I., Wang, C., van der Bliek, A.M., and Youle, R.J. (2014). Mitochondrial Rab GAPs govern autophagosome biogenesis during mitophagy. ELife *3*, e01612.

Yamashita, S., Oku, M., Wasada, Y., Ano, Y., and Sakai, Y. (2006). PI4P-signaling pathway for the synthesis of a nascent membrane structure in selective autophagy. J. Cell Biol. *173*, 709–717.

Yue, Z., and Zhong, Y. (2010). From a global view to focused examination: understanding cellular function of lipid kinase VPS34-Beclin 1 complex in autophagy. J. Mol. Cell Biol. *2*, 305–307.

Zaffagnini, G., and Martens, S. (2016). Mechanisms of Selective Autophagy. J. Mol. Biol. *428*, 1714–1724.

Zewe, J.P., Wills, R.C., Sangappa, S., Goulden, B.D., and Hammond, G.R. (2018). SAC1 degrades its lipid substrate PtdIns4*P* in the endoplasmic reticulum to maintain a steep chemical gradient with donor membranes. eLife *7*, e35588.

Zewe, J.P., Miller, A.M., Sangappa, S., Wills, R.C., Goulden, B.D., and Hammond, G.R.V. (2020). Probing the subcellular distribution of phosphatidylinositol reveals a surprising lack at the plasma membrane. J. Cell Biol. *219*, e201906127.

Zhang, H., Zhou, J., Xiao, P., Lin, Y., Gong, X., Liu, S., Xu, Q., Wang, M., Ren, H., Lu, M., et al. (2020). PtdIns4P restriction by hydrolase SAC1 decides specific fusion of autophagosomes with lysosomes. Autophagy, 1–11.

Zoncu, R., Bar-Peled, L., Efeyan, A., Wang, S., Sancak, Y., and Sabatini, D.M. (2011). mTORC1 senses lysosomal amino acids through an inside-out mechanism that requires the vacuolar H(+)-. ATPase Science *334* (*6056*), 678–83.

STAR★METHODS

KEY RESOURCES TABLE

REAGENT or RESOURCE	SOURCE	IDENTIFIER
**Antibodies**		
Rabbit anti-LC3 clone APG8C	Sigma-Aldrich	SAB1301850
Rabbit anti-LC3B	Cell Signaling Technology	3868
Mouse anti-β-Actin	Sigma-Aldrich	A5441; RRID:AB_476744
Rabbit anti-NDP52	Abcam	ab68588; RRID:AB_1640255
Mouse anti-p62	Abcam	ab109012; RRID:AB_2810880
Mouse anti-V5	Abcam	ab27671; RRID:AB_471093
FK2 anti-ubiquitin	Enzo Life Sciences	BML-PW8810; RRID:AB_10541840
Rabbit anti-SACM1L	Abnova	H00022908-D01P; RRID:AB_10632266
Rabbit anti-SACM1L	Thermo Fisher	13033-1-AP; RRID:AB_2301284
Mouse anti-TOMM20	Santa Cruz Biotechnology	sc-17764; RRID:AB_628381
Alexa Fluor-conjugated secondary antibodies	Thermo Fisher	A31571; RRID:AB_162542, A21206; RRID:AB_2535792, A32744; RRID:AB_2762826
Sheep anti-TGN46	Bio-Rad	AHP500; RRID:AB_324049
Mouse anti-GM130	BD	610822; RRID:AB_398141
Mouse anti-WIPI2	Abcam	ab105459; RRID:AB_10860881
Mouse anti-PtdIns(4)P IgM	Echelon	Z-P004-2
Rabbit anti-LAMP1	Cell Signaling Technology	9091; RRID:AB_2687579
**Bacterial and virus strains**		
S. Typhimurium SL1344	Hoiseth and Stocker, 1981	N/A
S. Typhimurium SL1344 Xen26	Conway et al., 2013	N/A
S. Typhimurium SL1344 DsRed	Rioux et al., 2007	N/A
S. Typhimurium SL14028s Δ*steA*	Laboratory of Dr. Luís Jaime Mota	N/A
S. Typhimurium SL14028s Δ*steA* reconstituted with PsteA-SteA	This study	N/A
S. Typhimurium SL14028s Δ*steA* reconstituted with PrpsM-SteA	This study	N/A
S. Typhimurium SL14028s Δ*steA* reconstituted with PsteA-SteA K36A	This study	N/A
S. Typhimurium SL14028s Δ*steA* reconstituted with PsteA-SteA-V5	This study	N/A
S. Typhimurium SL1344 x-light-mCherry	This study	N/A
S. Typhimurium SL1344 mCerulean	This study	N/A
**Chemicals, peptides, and recombinant proteins**		
Torin1	MedChemExpress	Hy-13003
Bafilomycin A1	Selleckchem	S1413
Hoechst 33342	Thermo Fisher	H3570
Puromycin	InvivoGen	ant-pr-1
Isopropyl β-D-1-thiogalactopyranoside	Sigma-Aldrich	367-93-1
Chloroquine	Sigma-Aldrich	C6628
Carbonyl cyanide 3-chlorophenylhydrazone (CCCP)	Sigma-Aldrich	C2759
Oligomycin	Sigma-Aldrich	75351
Antimycin A	Sigma-Aldrich	A8674
Lipofectamine 2000	Thermo Fisher	11668027

(*Continued on next page*)

Continued

REAGENT or RESOURCE	SOURCE	IDENTIFIER
Lipofectamine RNAiMAX	Thermo Fisher	13778150
Paraformaldehyde	Electron Microscopy Sciences	15714S
VectaShield	Vector Laboratories	H-1000-10
Critical commercial assays		
LysoTracker Red DND-99	Thermo Fisher	L7528
DQ Green BSA	Thermo Fisher	D12050
Ovalbumin AF647	Thermo Fisher	O34784
CellTracker Deep Red Dye	Thermo Fisher	C34565
BODIPY FL-pepstatin A	Thermo Fisher	P12271
Magic Red Cathepsin B	ImmunoChemistry Technologies	938
LysoView 633	Biotium	70058
Gibson Assembly Master Mix	New England Biolabs	E2611
Experimental models: Cell lines		
Human: HeLa	ATCC	Cat #CCL-2; RRID:CVCL_0030
Human: HeLa *SACM1L* KO	This paper	N/A
Human: HeLa *SACM1L* KO reconstituted with BFP-V5	This paper	N/A
Human: HeLa *SACM1L* KO reconstituted with *SACM1L*-V5 WT	This paper	N/A
Human: HeLa *SACM1L* KO reconstituted with *SACM1L*-V5 C389S	This paper	N/A
Human: HeLa *NDP52* KO	This paper	N/A
Human: HeLa mCherry-GFP-LC3	This paper	N/A
Human: HeLa GFP-LC3	This paper	N/A
Human: HeLa GFP-LC3; LAMP1-mCherry	This paper	N/A
Human: HeLa GFPLC3; BFP-2xP4M	This paper	N/A
Human: HeLa *SACM1L* KO mCherry-GFP-LC3	This paper	N/A
Human: HeLa *SACM1L* KO GFP-LC3	This paper	N/A
Human: HeLa *SACM1L* KO GFP-LC3; LAMP1-mCherry	This paper	N/A
Human: HeLa *SACM1L* KO GFP-LC3; BFP-2xP4M	This paper	N/A
Human: H4	ATCC	Cat#HTB-148; RRID:CVCL_1239
Oligonucleotides		
Silencer Select siRNA	Thermo Fisher	See Table S2
Recombinant DNA		
CGSW-mCherry-GFP-LC3	Laboratory of Dr. Christian Münz	N/A
CSGW-GFP-LC3	Laboratory of Dr. Christian Münz	N/A
pKB269 IPTG inducible x-light-mCherry	Laboratory of Dr. Serge Mostowy	Sirianni et al., 2016
pBMN-mCherry-Parkin	Addgene	(Yamano et al., 2014); RRID:Addgene_59419
pEGFP-hGal3	Addgene	Maejima et al., 2013; RRID:Addgene_73080
LAMP1-mRFP-FLAG	Addgene	(Zoncu et al., 2011); RRID:Addgene_34611
pFPV25.1	Addgene	Valdivia and Falkow, 1996
pFPV25.1_rpsM_mCerulean3	Addgene	Lane et al., 2019; RRID:Addgene_124904

(*Continued on next page*)

Continued

REAGENT or RESOURCE	SOURCE	IDENTIFIER
pXPR-BRD023	Broad Institute	N/A
pXPR-BRD003	Broad Institute	N/A
pLX317-BFP	Broad Institute	N/A
pLX317-SACM1L-V5	This paper	N/A
pLX317-SACM1L(C389S)-V5	This paper	N/A
pSFFV-P4M(SidM)x2-BFP	This paper	N/A
pFPV25.1-PrpsM-SteA	This paper	N/A
pFPV25.1-PSteA-SteA	This paper	N/A
pFPV25.1-PsteA-SteA(K36A)	This paper	N/A
pFPV25.1-PSteA-SteA-V5	This paper	N/A
pFPV25.1-PsteA-SteA(K36A)-V5	This paper	N/A
pLX304-EF1a-SteA-V5	This paper	N/A
Software and algorithms		
NIS-Elements	Nikon	N/A
Harmony High-Content Imaging and Analysis Software	Perkin Elmer	N/A
GraphPad Prism8	GraphPad Software, Inc.	N/A
Flowjo	FLOWJO	https://www.flowjo.com/

RESOURCE AVAILABILITY

Lead contact

Further information and requests for resources and reagents should be directed to and will be fulfilled by the Lead Contact, Ramnik J. Xavier (xavier@molbio.mgh.harvard.edu).

Materials availability

Materials generated in this study will be provided upon request.

Data and code availability

- The published article contains all datasets generated during this study.
- This paper does not report original code.
- Any additional information required to reanalyze the data reported in this paper is available from the lead contact upon request.

EXPERIMENTAL MODEL AND SUBJECT DETAILS

Cell line culture and small interfering RNA (siRNA) knockdown

HeLa and HEK293 cells were cultured at 37°C with 5% CO_2 in Dulbecco's modified Eagle's medium (DMEM) supplemented with 10% fetal bovine serum (FBS) (Sigma Aldrich), 100U/ml penicillin and 100mg/ml streptomycin. Transfections were performed with Lipofectamine 2000 according to the manufacturer's instructions. siRNA knockdowns were achieved by transfection with Lipofectamine RNAiMAX according to the manufacturer's instructions using Silencer Select siRNAs at 2nM (Table S2). For the siRNA knockdown screen of lipid kinase and phosphatase genes, HeLa cells were transfected with siRNAs at 2nM for 24h, followed by a media change and cultured for another 36h prior to infection with *S*. Typhimurium SL1344 expressing luciferase.

All the stable cell lines were generated with lentiviral transduction followed by either antibiotic selection or flow cytometry sorting based on the fluorescent tags.

Vector construction

All the plasmids in this study were generated by PCR and Gibson cloning using standard protocols with Gibson Assembly Master Mix.

Bacterial strains

Salmonella enterica serovar Typhimurium SL1344 and 14028s were grown in Luria-Bertani (LB) liquid media or on LB agar plates. *S*. Typhimurium Δ*steA* deletion in 14028s strain was a gift from Dr. Luís Jaime Mota (Domingues et al., 2014). Reconstituted strains were generated using standard transformation procedures (Figueira et al., 2013) and grown at 37°C on LB plates containing the appropriate antibiotic for selection.

METHOD DETAILS

Generation of CRISPR knockout cells

The Cas9 vector (pXPR_BRD023, Broad Institute) containing a *SACM1L*-specific single guide RNA (sgRNA) sequence was used to transfect HeLa cells using Lipofectamine 2000 according to the manufacturer's instructions. Media was replaced 24h post-transfection with selection media containing puromycin (2μg/ml). After 48h selection, surviving cells were plated in 96-well plates at 0.5 cells/well to isolate single clones. *SACM1L* knockout was confirmed by western blot and next generation sequencing. Guide RNA sequences:

SACM1L sg1: ACUGGGCACAAUCCAUCUGG
SACM1L sg2: UGGCUGUAAAAUACCUGCAA

Bacterial infection assays

Intracellular bacterial replication of *S.* Typhimurium SL1344 or SL1344 expressing bacterial luciferase was measured by a colony-forming unit (CFU) or luciferase assay, respectively. In general, *S.* Typhimurium strains SL1344 or 14028s were grown overnight from a single colony then subcultured for 3h at 37°C until late log phase. Subcultures were diluted in complete DMEM media containing 10% FBS to achieve multiplicity of infection (MOI) of 100:1. Cells were infected with bacteria for 30min at 37°C, rinsed twice then incubated with complete DMEM media supplemented with 50 μg/ml gentamicin (Thermo Fisher) for 1h to kill extracellular bacteria. Cells were then rinsed twice and cultured with complete DMEM media containing 20μg/ml gentamicin. For luciferase assays, the first luciferase reading was taken at 1.5h post-infection and then every hour until 9.5h post-infection. For CFU assays, HeLa cells were lysed with 1% Triton X-100 at room temperature (RT) for 10min at indicated time points and intracellular *Salmonella* were serially diluted, plated on LB plates and colonies were counted following overnight incubation at 30°C. For both assays, the fold change was determined relative to the initial measured value at 1.5h post-infection.

For indicated imaging experiments, bacteria were labeled with CellTracker Deep Red Dye. Bacterial subcultures were washed three times with phosphate buffered saline (PBS) and incubated with 5 μM CellTracker Deep Red Dye for 30min at 37°C with gentle agitation. Excessive dye was quenched by washing with Super Optimal Broth (SOB) media twice. Bacteria were recovered in SOB media for 30min at 37°C with gentle agitation. After the final wash with SOB media, the bacteria were used for infection as above.

Chloroquine (CHQ) resistance assay

CHQ resistance assay was performed as described previously (Knodler et al., 2014). HeLa cells were infected as in the CFU assay described above. One hour prior to the 1.5- and 7.5-hour time points, cells were treated with CHQ (200 μM) and gentamicin (50 μg/ml for the 1.5-hour time point; 20 μg/ml for 7.5-hour time point) for 1h. Control cells not treated with CHQ were incubated with gentamicin only. At each time point post-infection, cytosolic replication of *Salmonella* was evaluated as described above in the bacterial CFU assay.

X-light-mCherry bacterial assay

Cells were infected with bacteria expressing an IPTG-inducible fluorescent protein (x-light-mCherry) as an indicator of metabolic activity at the time of IPTG addition (Krokowski et al., 2018). For each time point, prior to fixation, the cells were treated with 1.5mM IPTG for 45min in complete DMEM media at 37°C, then fixed and stained for imaging.

Immunofluorescence

Cells were seeded and grown on sterile glass coverslips or in a 96-well plate. Following treatments, cells were fixed and permeabilized with either ice cold 100% methanol for 3min or with PBS containing 4% (v/v) paraformaldehyde (PFA) for 20min followed by PBS containing 0.2% (w/v) saponin for 8min. After extensive washing with PBS, coverslips were incubated in blocking buffer [PBS containing 5% (v/v) normal goat serum and 0.05% (w/v) saponin] for 1h at RT and then incubated with primary antibodies in the same buffer at 4°C overnight. Cells were washed with PBS then incubated with Alexa Fluor-conjugated secondary antibodies in blocking buffer for 1h at RT. After washing with PBS, cells were sealed with Vectashield mounting medium for confocal microscopy analysis as described below.

Immunostaining of endogenous phosphatidylinositol 4-phosphate [PI(4)P] was performed as previously described (Hammond et al., 2009) with the following modifications. Briefly, cells were fixed for 15min with 0.1M phosphate buffer, pH 7.4 (PB) containing 2% (v/v) PFA then washed three times with PBS containing 50mM NH_4Cl. Cells were permeabilized for 5min with 20 μM digitonin in buffer A [20mM PIPES (pH 6.8) containing 137mM NaCl and 2.7mM KCl] then blocked for 1h with blocking buffer (Buffer A containing 5% normal goat serum and 50mM NH_4Cl). Cells were incubated with an anti-PI(4)P primary antibody diluted 1:200 in buffer A for 1h at RT or 4°C overnight. After two washes with buffer A, secondary antibodies were added for 45min in the blocking buffer. Cells were washed and post-fixed for 5min with PBS containing 2% (v/v) PFA. Fixative was removed by washing three times with PBS containing 50mM NH_4Cl then subjected to imaging.

Staining of BODIPY FL-pepstatin A was performed after secondary antibodies. Cells were washed twice with 300mM sodium acetate (pH 4.5) with 0.1% Brij 35, then incubated in the same buffer containing 1uM BODIPY FL-pepstatin A at RT for 1h. Cells were washed again with sodium acetate buffer before imaging.

BFP-2xP4M and GFP-LC3 were stably expressed by lentiviral transduction. After 7d, cells were infected with DsRed-expressing *Salmonella*, washed and imaged by live confocal microscopy at 2h post-infection.

Microscopy

For fixed cells, fluorescence images were obtained with a Perkin Elmer Opera Phenix system using a 60x 1.42 N.A. water objective or with a Nikon Ti2-E inverted microscope equipped with a CSU-W1 spinning disc confocal and Andor Zyla 4.2 sCMOS camera using a 100x 1.40 N.A. oil objective. To record the dynamics of xenophagy in HeLa cells, images were obtained with the Perkin Elmer Opera Phenix system using a 60x 1.42 N.A. water objective. Cells were plated in CellCarrier-96 Ultra Microplates in DMEM supplemented with 10% FBS then treated and infected as indicated. During imaging, the plates or dishes were placed in a humidified chamber supplemented with 5% CO_2 at 37°C. Images were captured every 15 or 30min with a Z stack of 1 μm/section for a total of three sections. After acquisition, the images were projected to form one image by maximum-intensity projection. Analysis of images was performed using Harmony High-Content Imaging and Analysis Software or Columbus Image Data Storage and Analysis System. To quantify association of markers with intracellular bacteria, we defined the bacterial region using the micronuclei software setting, which was expanded by 2 pixels beyond the bacterial signal to detect overlapping signals in a defined and consistent manner. The mean and sum intensities as well as contrast of associated fluorescent signals within the bacterial region were calculated and used to define a threshold for each of the individual markers being evaluated. All parameters were unchanged when quantifying the percentage of association in WT and *SACM1L* KO cells.

Quantitative PCR

Total RNA was isolated using the RNeasy Mini kit (QIAGEN, Waltham, MA). cDNA was prepared using 0.5-1 μg total RNA by RT-PCR using iScript cDNA synthesis kit (Bio-Rad, Hercules, CA) according to manufacturer's instructions. qPCR was performed on 5 μL cDNA using iTaq Universal SYBR Green Supermix (Bio-Rad) and primers designed to recognize the indicated genes. Fold changes were calculated by the Delta-Delta-Ct method using human GAPDH as the control. All fold changes were expressed as normalized to the WT or untreated control.

QUANTIFICATION AND STATISTICAL ANALYSIS

For analysis of the siRNA screen, data was normalized to the SiSel_NC control siRNA on each plate and log_2 transformed. For each gene, a linear mixed-effects model (lme function in nlme R package) was then used to test whether the log expression difference from SiSel_NC was nonzero, with the experiment as the random effect. P values were derived from F tests. Multiple hypothesis correction was done using the Benjamini-Hochberg false discovery rate.

Statistical analyses were done using Prism (GraphPad Software) or Excel (Microsoft Office) to generate curves or bar graphs. All error bars represent standard error of the mean (SEM). Two-tailed unpaired t tests were used for statistical analysis of two groups of samples. One-way ANOVA analysis of variance with a Newman-Keuls post-test was used to evaluate statistical significance of multiple groups of samples. *$p < 0.05$; **$p < 0.01$; ***$p < 0.001$, ****$p < 0.0001$. $p \geq 0.05$ was considered not significant (NS).

Cell Reports, Volume *36*

Supplemental information

SAC1 regulates autophagosomal phosphatidylinositol-4-phosphate for xenophagy-directed bacterial clearance

Kai Liu, Lingjia Kong, Daniel B. Graham, Kimberly L. Carey, and Ramnik J. Xavier

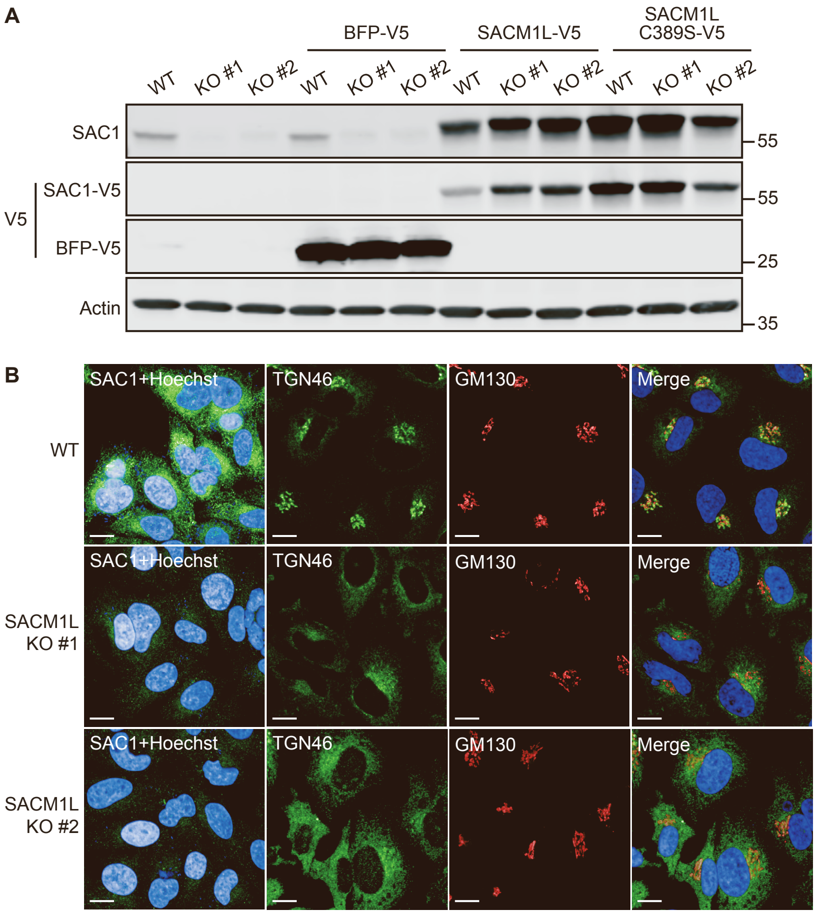

Figure S1. Validated SAC1-deficient cell lines have dispersed trans-Golgi network (TGN), Related to Figure 1. A) Representative immunoblot of WT and *SACM1L* KO cells with or without stable expression of V5-tagged BFP, SAC1 WT, or phosphatase inactive SAC1 C389S. Cells were reconstituted by lentiviral transduction and selected with puromycin for 7 days before the experiment. Endogenous and re-expressed SAC1 were detected with an antibody against SAC1. Recombinantly expressed SAC1 WT, SAC1 C389S, and BFP were detected with an antibody against V5. Actin served as a loading control. Molecular weight markers are shown on the right. **B)** Knockout of *SACM1L* causes dispersed Golgi morphology. In representative confocal images, SAC1+Hoechst images demonstrate cytoplasmic localization of SAC1 in WT cells. No SAC1 was detected in *SACM1L* KO cells. Golgi and TGN were detected with antibodies against GM130 and TGN46, respectively, in WT and *SACM1L* KO cells. Merged images of GM130, TGN46 and Hoechst are shown. Scale bars represent 20μm. For all quantifications, over 500 cells were analyzed. Data from three independent experiments were analyzed using ANOVA (mean ± SEM).

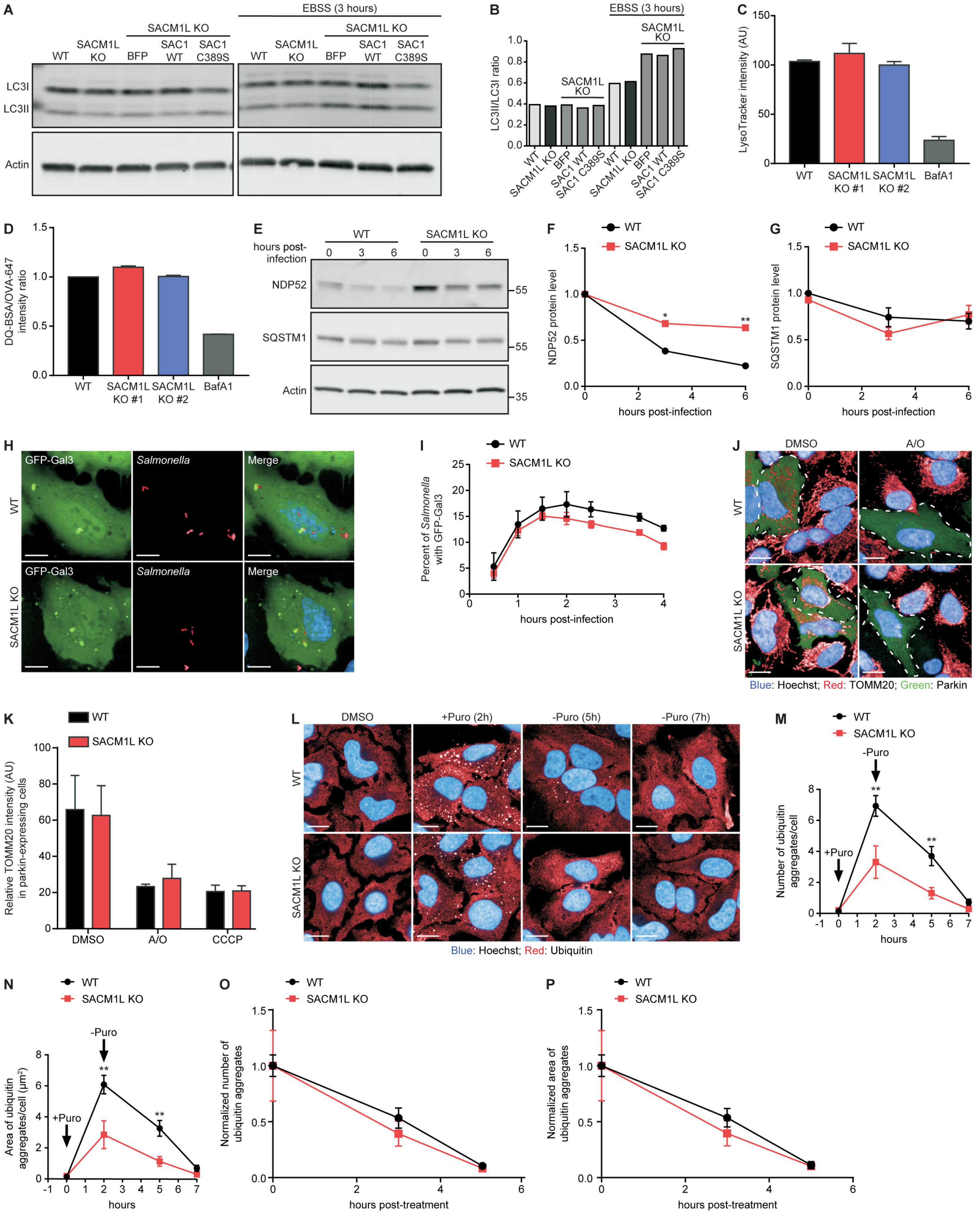
A
EBSS (3 hours)
SACM1L KO
WT
SACM1L KO
BFP
SAC1 WT
SAC1 C389S
LC3I
LC3II
Actin
B
EBSS (3 hours)
SACM1L KO
LC3II/LC3I ratio
1.0
0.8
0.6
0.4
0.2
0.0
WT
SACM1L KO
BFP
SAC1 WT
SAC1 C389S
C
LysoTracker intensity (AU)
150
100
50
0
WT
SACM1L KO #1
SACM1L KO #2
BafA1
D
DQ-BSA/OVA-647 intensity ratio
1.5
1.0
0.5
0.0
WT
SACM1L KO #1
SACM1L KO #2
BafA1
E
hours post-infection
WT
SACM1L KO
0
3
6
NDP52
SQSTM1
Actin
55
55
35
F
WT
SACM1L KO
NDP52 protein level
1.5
1.0
0.5
0.0
*
**
0
2
4
6
hours post-infection
G
WT
SACM1L KO
SQSTM1 protein level
1.5
1.0
0.5
0.0
0
2
4
6
hours post-infection
H
GFP-Gal3
Salmonella
Merge
WT
SACM1L KO
I
WT
SACM1L KO
Percent of Salmonella with GFP-Gal3
25
20
15
10
5
0
0
1
2
3
4
hours post-infection
J
DMSO
A/O
WT
SACM1L KO
Blue: Hoechst; Red: TOMM20; Green: Parkin
K
WT
SACM1L KO
Relative TOMM20 intensity (AU) in parkin-expressing cells
100
80
60
40
20
0
DMSO
A/O
CCCP
L
DMSO
+Puro (2h)
-Puro (5h)
-Puro (7h)
WT
SACM1L KO
Blue: Hoechst; Red: Ubiquitin
M
WT
SACM1L KO
-Puro
+Puro
**
**
Number of ubiquitin aggregates/cell
8
6
4
2
0
-1
0
1
2
3
4
5
6
7
hours
N
WT
SACM1L KO
-Puro
+Puro
**
**
Area of ubiquitin aggregates/cell (µm²)
8
6
4
2
0
-1
0
1
2
3
4
5
6
7
hours
O
WT
SACM1L KO
Normalized number of ubiquitin aggregates
1.5
1.0
0.5
0.0
0
2
4
6
hours post-treatment
P
WT
SACM1L KO
Normalized area of ubiquitin aggregates
1.5
1.0
0.5
0.0
0
2
4
6
hours post-treatment

Figure S2. Loss of SAC1 activity has no effect on bulk autophagy, mitophagy or aggrephagy, Related to Figure 2. A-B) Representative immunoblot (A) and quantification (B) of LC3 conversion in WT, *SACM1L* KO and reconstituted cells grown in complete or amino acid-deficient (EBSS) media for 3 hours to induce non-selective autophagy. Actin served as a loading control. **C-D)** *SACM1L* KO cells have normal lysosomal function. FACS quantification of LysoTracker intensity (AU, arbitrary units) (C) or DQ-Green BSA degradation activity (D) in WT and *SACM1L* KO cells. WT cells were treated with BafA1 (200ng/ml) for 4 hours as a control for defective lysosomal acidification and degradation. Cells in (D) were treated with DQ-BSA (10µg/ml), OVA-647 (5µg/ml) and EGF (50ng/ml) for 1 hour, washed then cultured for 30 minutes before FACS analysis. **E-G)** Delayed NDP52 degradation in *SACM1L* KO cells after *Salmonella* infection. Representative immunoblot (E) and quantification of NDP52 (F) and SQSTM1 (G) protein levels in WT and *SACM1L* KO cells at indicated time points post-infection. Protein levels were normalized to the start of infection for each genotype. Actin served as a loading control. **H-I)** SAC1 does not affect Galectin 3 dynamics on *Salmonella*-containing vacuoles. Representative confocal images (H) and quantification (I) of *Salmonella* associated with GFP-Gal3 in WT and *SACM1L* KO cells. Cells transiently expressing GFP-Gal3 were infected with DsRed-expressing *Salmonella*. Following gentamicin treatment, cells were imaged by live confocal microscopy. (H) Representative images at 1 hour post-infection. Scale bars represent 10µm. (I) Quantification of the percentage of *Salmonella* co-localized with GFP-Gal3 at indicated time points post-infection. **J-K)** Loss of SAC1 does not affect Parkin-dependent mitophagy. Representative confocal images (J) and quantification (K) of TOMM20 in WT and *SACM1L* KO cells transiently expressing mCherry-Parkin. Following transfection, cells were treated with DMSO, CCCP (20µM) or a combination of oligomycin (5µM) and antimycin A (2µM), indicated as A/O, for 24 hours. Images are pseudo-colored such that endogenous TOMM20, Parkin and Hoechst are shown in red, green and blue, respectively. Quantification represents intensity of TOMM20 signal in Parkin$^+$ cells within the dashed lines in images (J). Scale bars represent 20µm. **L-P)** Loss of SAC1 does not affect aggrephagy. Representative confocal images (L) and quantification (M-P) of ubiquitin immunostaining in WT and *SACM1L* KO cells. Cells were treated with puromycin (5µM) for 2 hours to induce formation of protein aggregates, washed then cultured for 3 hours or 5 hours before immunostaining. Quantification represents the number (M) and area (N) of ubiquitin$^+$ aggregates per cell at indicated time points. Normalized number (O) and area (P) of ubiquitin$^+$ aggregates was determined by dividing the data at the indicated time point by the data after 2 hours of puromycin treatment. Scale bars represent 20µm. For all quantifications, over 200 cells were analyzed. Data from three independent experiments were analyzed using ANOVA (mean ± SEM). *$p<0.05$, **$p<0.01$.

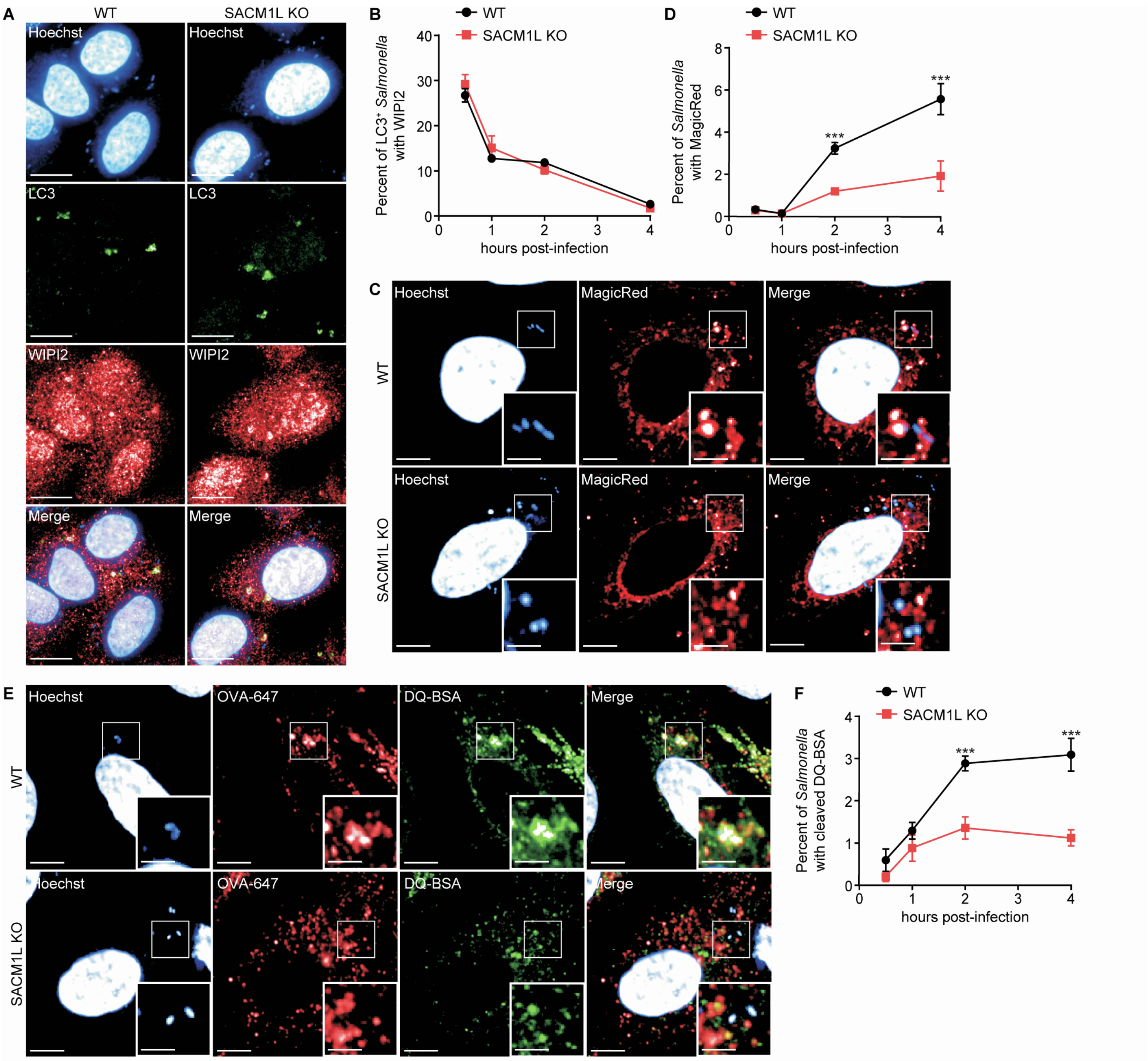

Figure S3. Loss of SAC1 delays delivery of lysosomal enzymes, not autophagosome closure, Related to Figure 3. A-B) WIPI2 recruitment and dissociation from *Salmonella*-containing autophagosomes are not affected by SAC1. Representative confocal images (A) and quantification (B) of *Salmonella* associated with WIPI2 in WT and *SACM1L* KO cells. Cells were fixed and imaged at indicated time points post-infection. A) Representative images of the infected cells at 1 hour post-infection. Scale bars represent 20μm. B) Percentage of LC3$^+$ *Salmonella* that are also WIPI2$^+$ at indicated time points post-infection. **C-D)** Delivery of cathepsin B to *Salmonella*-containing autophagosomes is delayed by SAC1 depletion. Representative confocal images (C) and quantification (D) of *Salmonella* associated with MagicRed in WT and *SACM1L* KO cells. Cells were pretreated with MagicRed for 30 minutes before infection, fixed and imaged at indicated time points post-infection. C) Representative images of the infected cells at 2 hours post-infection. Insets show magnified (2x) views of the boxed region in each image. Scale bars represent 10μm in full images and 5μm in insets. D) Percentage of MagicRed$^+$ *Salmonella* at indicated time points post-infection. **E-F)** Delivery of lysosome hydrolases to *Salmonella*-containing autophagosomes is delayed by SAC1 depletion. Representative confocal images (E) and quantification (F) of *Salmonella* associated with DQ-Green BSA and OVA-647 in WT and *SACM1L* KO cells. Cells were pretreated with DQ-Green BSA (10μg/ml) and OVA-647 (5μg/ml) for 1 hour before being infected, fixed and imaged at indicated time points post-infection. E) Representative images of the infected cells at 2 hours post-infection. Insets show magnified (2x) views of the boxed region in each image. Scale bars represent 10μm in full images and 5μm in insets. F) Percentage of DQ-Green BSA$^+$ *Salmonella* at indicated time points post-infection. For all quantifications, over 500 cells were analyzed. Data from at least two independent experiments were analyzed using ANOVA (mean ± SEM). ***$p<0.001$.

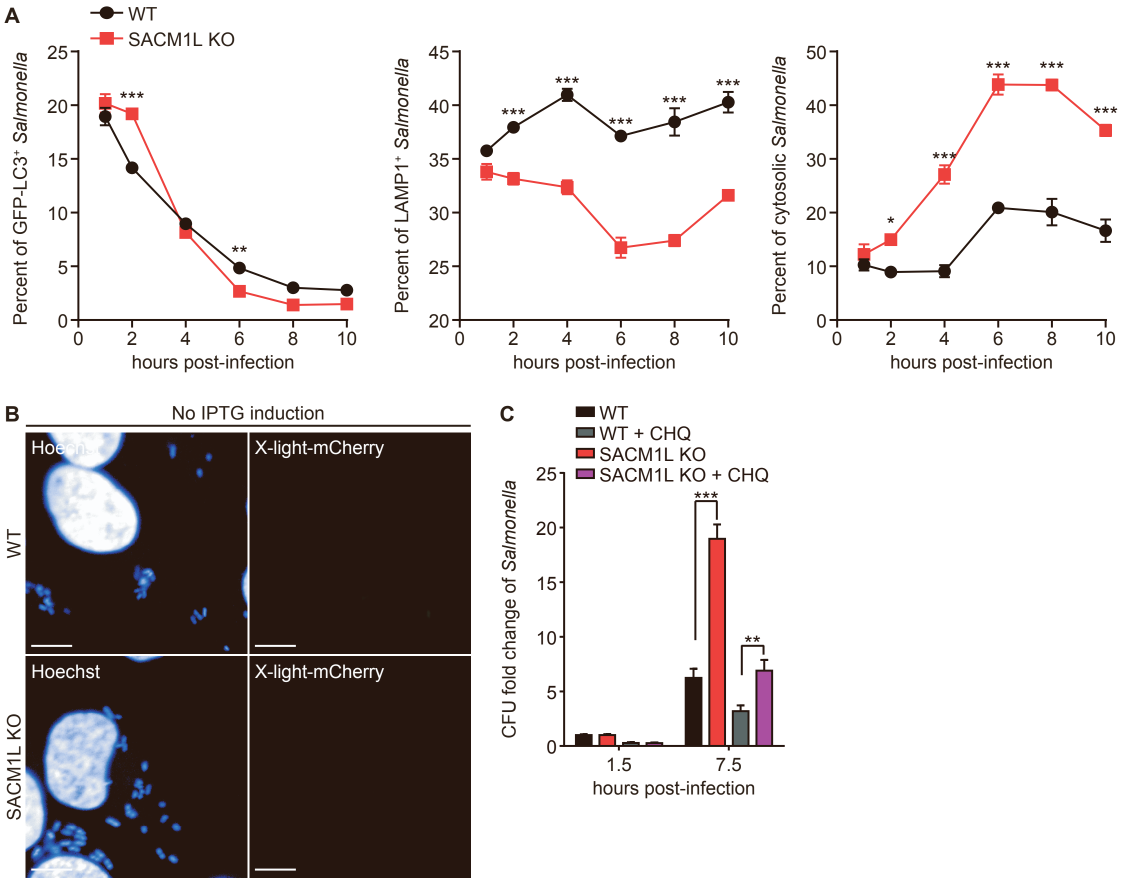

Figure S4. Delayed autophagosome-lysosomal fusion results in increased cytosolic bacteria, Related to Figure 4. A) Percentage of GFP-LC3$^+$, LAMP1$^+$ or GFP-LC3$^-$LAMP1$^-$ (cytosolic) *Salmonella* in WT and *SACM1L* KO cells at indicated time post-infection. Quantification of GFP-LC3$^-$LAMP1$^-$ *Salmonella* populations excludes bacteria with LC3 or LAMP1 signal in proximity to make the data more precise. Over 5,000 bacteria were analyzed. **B)** Representative confocal images of *Salmonella* expressing x-light-mCherry without IPTG induction in WT and *SACM1L* KO cells at 6 hours post-infection. Scale bars represent 10µm. **C)** Contribution of non-SCV bacteria to increased intracellular replication in *SACM1L* KO cells. Infected cells were treated with 200 µM chloroquine (CHQ) for 1 hour prior to lysis and plating for CFU. Fold change of CFU was normalized to the 1.5-hour time point at indicated time points post-infection. For all quantifications, over 3,000 cells and 5,000 bacteria were analyzed. Data from at least three independent experiments were analyzed using ANOVA (mean ± SEM). *p<0.05, **p<0.01, ***p<0.001.

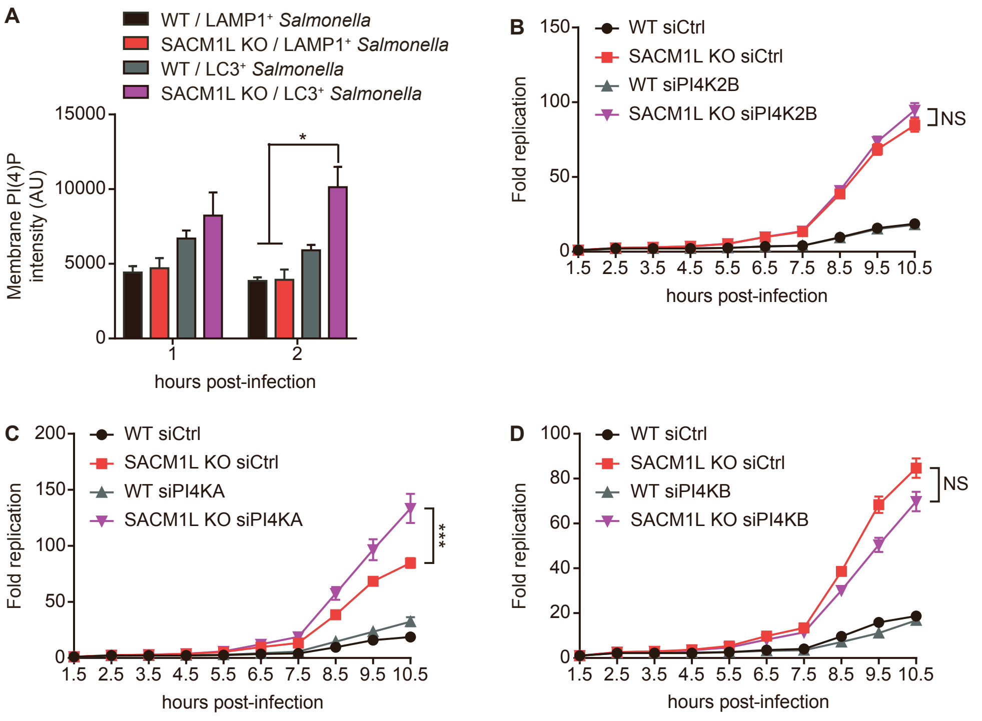

Figure S5. Defect in xenophagy due to loss of SAC1 is not rescued by kinase expression, Related to Figure 5 and Table S2. A) Quantification of the PI(4)P staining intensity (AU, arbitrary units) on LC3+ or LAMP1+ *Salmonella* in WT and *SACM1L* KO cells at indicated time points post-infection. **B-D)** For quantifications in knockdown experiments, WT or *SACM1L* KO cells were transfected with siRNA for control (Ctrl) or indicated genes. After 48 hours, cells were infected with *Salmonella* SL1344 expressing luciferase, and luciferase levels were measured over time. Bacterial replication was normalized to the baseline infection level. Knockdowns of PI4K2B (B), PI4KA (C) or PI4KB (D) do not affect *Salmonella* replication in WT or *SACM1L* KO cells. Data from at least two independent experiments were analyzed using ANOVA (mean ± SEM). *p<0.05, ***p<0.001. NS, not significant.

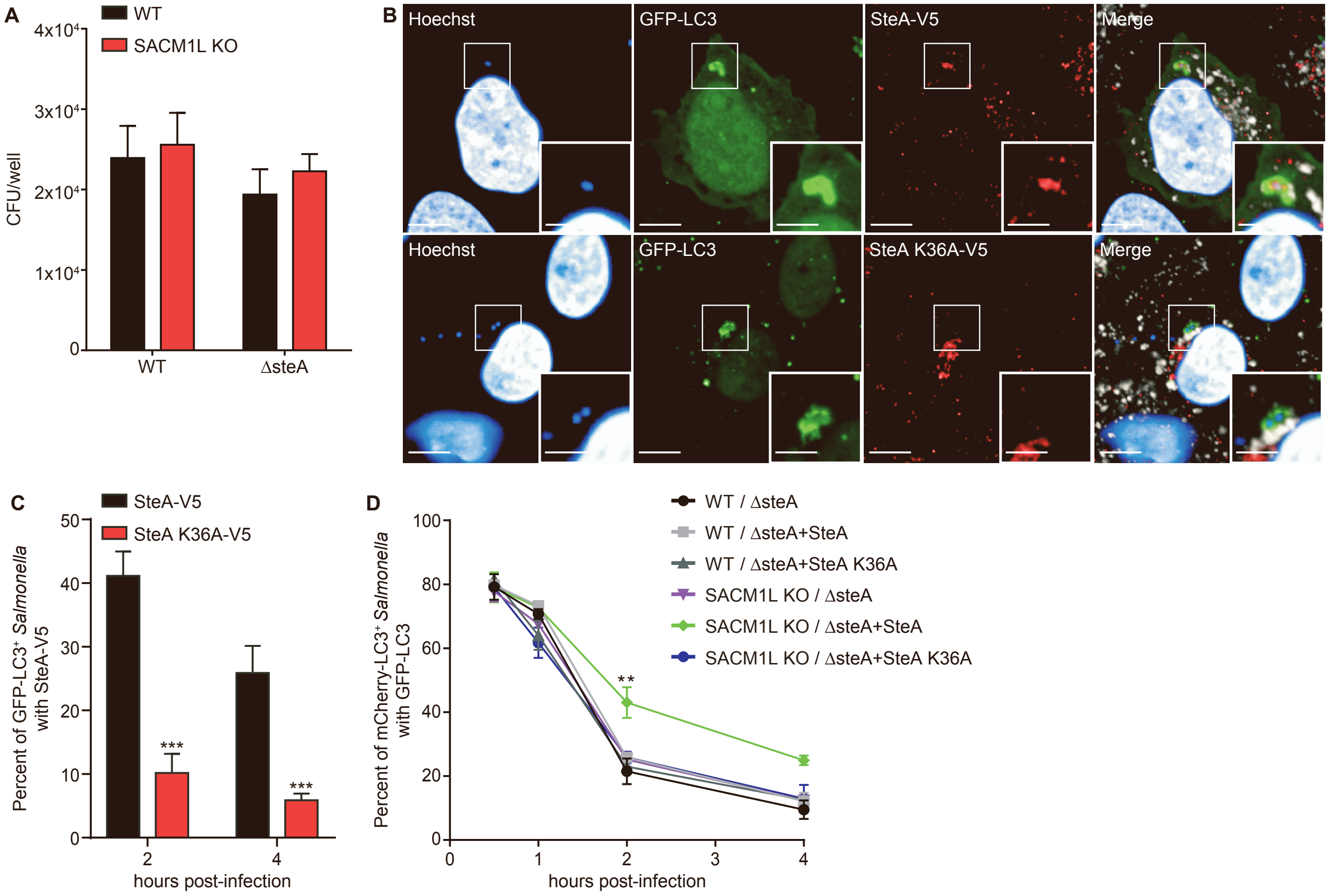

Figure S6. SteA PI(4)P binding is required to bind autophagosomes and block lysosomal fusion, Related to Figure 6. A) Neither loss of SAC1 nor *Salmonella* SteA affects bacterial uptake. Quantification of CFU/well of WT or Δ*steA Salmonella* at 1 hour after infection of WT or *SACM1L* KO cells. **B-C)** Subcellular location of *Salmonella*-secreted SteA or SteA K36A mutant. Representative confocal images (A) and quantification (B) of GFP-LC3$^+$ *Salmonella* associated with SteA-V5 or SteA K36A-V5 in WT cells. Cells were infected with the Δ*steA* mutant reconstituted with SteA-V5 or SteA K36A-V5, fixed and imaged at indicated time points post-infection. B) Representative images of infected WT cells at 2 hours post-infection. Insets show magnified (2x) views of the boxed region in each image. Scale bars represent 10µm in full images and 5µm in insets. C) Percentage of GFP-LC3$^+$ *Salmonella* with SteA-V5 or SteA K36A-V5 at indicated time points post-infection. Over 500 cells were analyzed. **D)** SteA is required to impair the maturation of *Salmonella*-containing autophagosomes. Percentage of mCherry-LC3$^+$ *Salmonella* also positive for GFP in WT and *SACM1L* KO cells at indicated times post-infection. Over 600 cells were analyzed. Data from at least two independent experiments were analyzed using ANOVA (mean ± SEM). $^{**}p<0.01$, $^{***}p<0.001$.
